# Constitutive nuclear accumulation of endogenous alpha-synuclein in mice causes motor impairment and cortical dysfunction, independent of protein aggregation

**DOI:** 10.1093/hmg/ddac035

**Published:** 2022-02-18

**Authors:** Haley M Geertsma, Terry R Suk, Konrad M Ricke, Kyra Horsthuis, Jean-Louis A Parmasad, Zoe A Fisk, Steve M Callaghan, Maxime W C Rousseaux

**Affiliations:** Department of Cellular and Molecular Medicine, University of Ottawa, Ottawa, ON K1H8M5, Canada; University of Ottawa Brain and Mind Research Institute, Ottawa, ON K1H8M5, Canada; Department of Cellular and Molecular Medicine, University of Ottawa, Ottawa, ON K1H8M5, Canada; University of Ottawa Brain and Mind Research Institute, Ottawa, ON K1H8M5, Canada; Department of Cellular and Molecular Medicine, University of Ottawa, Ottawa, ON K1H8M5, Canada; University of Ottawa Brain and Mind Research Institute, Ottawa, ON K1H8M5, Canada; Aligning Science Across Parkinson's (ASAP) Collaborative Research Network, Chevy Chase, MD; Department of Cellular and Molecular Medicine, University of Ottawa, Ottawa, ON K1H8M5, Canada; University of Ottawa Brain and Mind Research Institute, Ottawa, ON K1H8M5, Canada; Department of Cellular and Molecular Medicine, University of Ottawa, Ottawa, ON K1H8M5, Canada; University of Ottawa Brain and Mind Research Institute, Ottawa, ON K1H8M5, Canada; Department of Cellular and Molecular Medicine, University of Ottawa, Ottawa, ON K1H8M5, Canada; University of Ottawa Brain and Mind Research Institute, Ottawa, ON K1H8M5, Canada; Aligning Science Across Parkinson's (ASAP) Collaborative Research Network, Chevy Chase, MD; Department of Cellular and Molecular Medicine, University of Ottawa, Ottawa, ON K1H8M5, Canada; University of Ottawa Brain and Mind Research Institute, Ottawa, ON K1H8M5, Canada; Department of Cellular and Molecular Medicine, University of Ottawa, Ottawa, ON K1H8M5, Canada; University of Ottawa Brain and Mind Research Institute, Ottawa, ON K1H8M5, Canada; Aligning Science Across Parkinson's (ASAP) Collaborative Research Network, Chevy Chase, MD; Ottawa Institute of Systems Biology, University of Ottawa, Ottawa, ON K1H8M5, Canada; Eric Poulin Center for Neuromuscular Diseases, University of Ottawa Brain and Mind Research Institute, Ottawa, ON K1H8M5, Canada

## Abstract

A growing body of evidence suggests that nuclear alpha-synuclein (αSyn) plays a role in the pathogenesis of Parkinson’s disease (PD). However, this question has been difficult to address as controlling the localization of αSyn in experimental systems often requires protein overexpression, which affects its aggregation propensity. To overcome this, we engineered *Snca^NLS^* mice, which localize endogenous αSyn to the nucleus. We characterized these mice on a behavioral, histological and biochemical level to determine whether the increase of nuclear αSyn is sufficient to elicit PD-like phenotypes. *Snca^NLS^* mice exhibit age-dependent motor deficits and altered gastrointestinal function. We found that these phenotypes were not linked to αSyn aggregation or phosphorylation. Through histological analyses, we observed motor cortex atrophy in the absence of midbrain dopaminergic neurodegeneration. We sampled cortical proteomes of *Snca^NLS^* mice and controls to determine the molecular underpinnings of these pathologies. Interestingly, we found several dysregulated proteins involved in dopaminergic signaling, including Darpp32, Pde10a and Gng7, which we further confirmed was decreased in cortical samples of the *Snca^NLS^* mice compared with controls. These results suggest that chronic *endogenous* nuclear αSyn can elicit toxic phenotypes in mice, independent of its aggregation. This model raises key questions related to the mechanism of αSyn toxicity in PD and provides a new model to study an underappreciated aspect of PD pathogenesis.

## Introduction

Alpha-synuclein (αSyn) is a protein notorious for its involvement in Parkinson’s disease (PD) pathogenesis. For one, it is a primary constituent of Lewy bodies and Lewy neurites, pathological hallmarks of PD (1). Moreover, copy number variations and missense mutations in the αSyn gene, *SNCA*, cause genetic forms of PD, further reinforcing its involvement in disease etiology (2–8). αSyn was first described as a presynaptic and nuclear protein (9). However, nuclear αSyn has largely been overshadowed by a focus on its cytoplasmic/synaptic form, likely due to the cytoplasmic localization of Lewy bodies. Despite this, several studies have linked nuclear αSyn to PD on multiple levels: in cell (10–14) and animal (6,15–18) models of PD, as well as in brain tissue from individuals with αSyn pathologies [synucleinopathy; (19–21)]. These studies examined the role of nuclear αSyn by overexpressing it together with mutations or a nuclear localization signal [NLS; (12,15,17,18,22,20)], or upon toxin exposure (16), hinting at a role for nuclear αSyn in disease pathogenesis by its involvement in DNA binding (23,24) or histone modification (17) to alter transcription, and in DNA repair (25). Although these studies support a link between nuclear αSyn and PD, its specific role in disease—whether deleterious or beneficial—remains clouded due to the reliance on its overexpression or exogenous stressors, making it difficult to parse out the driver of toxicity in the absence of αSyn aggregation.

To directly test the consequence of chronic αSyn mislocalization to the nucleus *in vivo,* without resorting to protein overexpression, we engineered a mouse model to endogenously express αSyn with a C-terminal NLS-Flag tag driving its mislocalization to the nucleus. We extensively characterized these mice at the behavioral, histological and biochemical level to assess whether chronic nuclear localization of αSyn causes age-dependent phenotypes resembling PD or related αSyn proteinopathies.

## Results

### Effective nuclear targeting of αSyn in *Snca*^*NLS*^ mice

**Figure 1:**
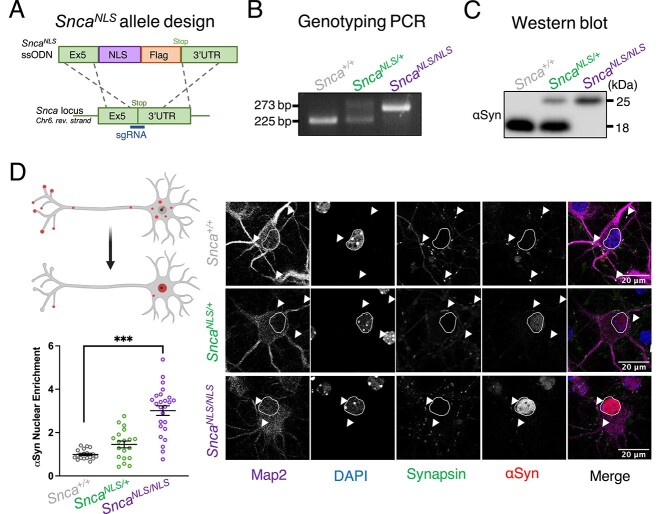
*Snca^NLS^* mice effectively target αSyn to the nucleus *in vitro* and *in vivo*. (**A**) *Snca^NLS-Flag^* knock-in scheme with C-terminal NLS-Flag tag. Visualization of the knockin via (**B**) PCR and (**C**) western blot. (**D**) Illustration of nuclear localization of αSyn (upper left) with quantification (lower left) of nuclear αSyn from primary cortical neurons in wildtype (top right panels), *Snca^NLS/+^* (middle right panels) and *Snca^NLS/NLS^* (bottom right panels; *n* = 3). Protein quantification (lower left) depicts nested relative per-cell intensity of nuclear αSyn from three independent experiments. White arrows denote presynaptic αSyn and nuclei are circled in white. Nested one-way ANOVA with Bonferroni multiple comparison: ^**^denotes *P* < 0.001.

To study if nuclear αSyn is sufficient to elicit age-related behavioral and pathological phenotypes, we generated a mouse line that targets endogenous αSyn to the nucleus via the knockin of an NLS-Flag tag on αSyn (*Snca^NLS^*). The NLS-Flag construct was targeted to the 3′ end of the *Snca* coding sequence with the modified *Snca-NLS-Flag* gene predicted to transcribe a fusion protein of wildtype αSyn with a C-terminal NLS-Flag tag (Fig. 1A). After generating and backcrossing mice (see Materials and Methods and Supplementary Material, Fig. S1), we confirmed the knockin via sequencing (Supplementary Material, Fig. S1B) and were able to distinguish between the genotypes via a PCR band shift (Fig. 1B) and a larger protein size via western blot (Fig. 1C). Mice were born at expected Mendelian ratios (Supplementary Material, Fig. S1C), confirming that insertion of this tag did not pose major developmental deficits.

**Figure 2:**
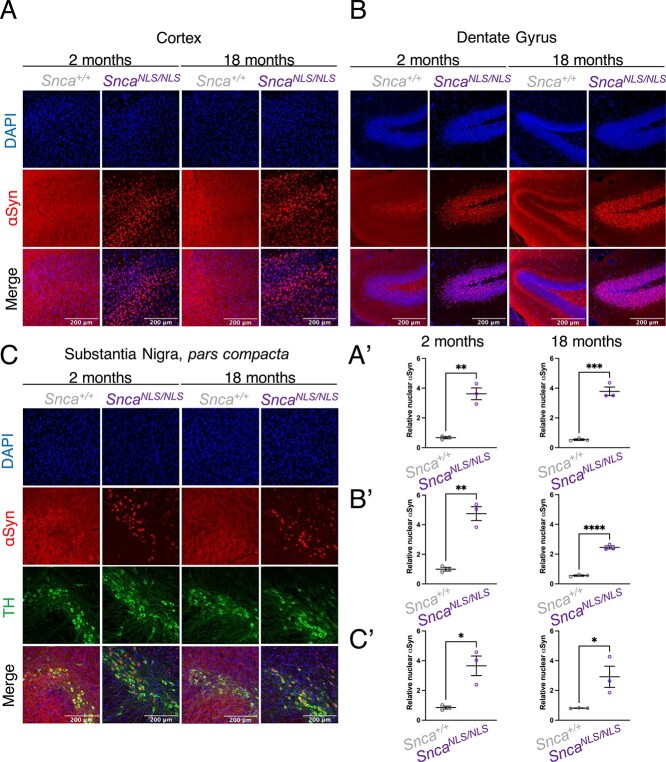
*Snca^NLS^* mice exhibit a consistent 2–5-fold increase in nuclear αSyn with age. Immunofluorescent images of 2- (left) and 18-month (right) mouse (**A**) cortex, (**B**) dentate gyrus and (**C**) substantia nigra, *pars compacta*. Nuclear enrichment of αSyn was quantified for (A′–C′) all brain regions at both 2- (left) and 18-months (right). Unpaired, two-tailed *t*-test: ^*^, ^**^, ^***^, ^****^ denote *P* < 0.05, < 0.01, < 0.001 and <0.0001, respectively.

To examine the efficiency of the NLS-Flag tag, we cultured primary cortical neurons for 7 days *in vitro* and quantified the level of nuclear αSyn through immunofluorescent microscopy. We observed a 3-fold increase in nuclear αSyn in *Snca^NLS/NLS^* and a 1.5-fold increase in *Snca^NLS/+^* compared with *Snca^+/+^* (wildtype) cells (Fig. 1D). This trend was consistent in stained adult mouse brain tissue at both 2- and 18-months where we found a 2–5-fold increase in nuclear αSyn in the cortex (Fig. 2A), dentate gyrus (Fig. 2B) and substantia nigra (Fig. 2C). Importantly, this roughly corresponds to the 2.5–3-fold increase of nuclear αSyn, which we and others have previously observed in post-mortem brain tissue from individuals with PD or other animal models of synucleinopathy, suggesting the model displays a disease-relevant increase of nuclear αSyn (6,15–19).

### Increased nuclear αSyn leads to an age-dependent motor decline

**Figure 3:**
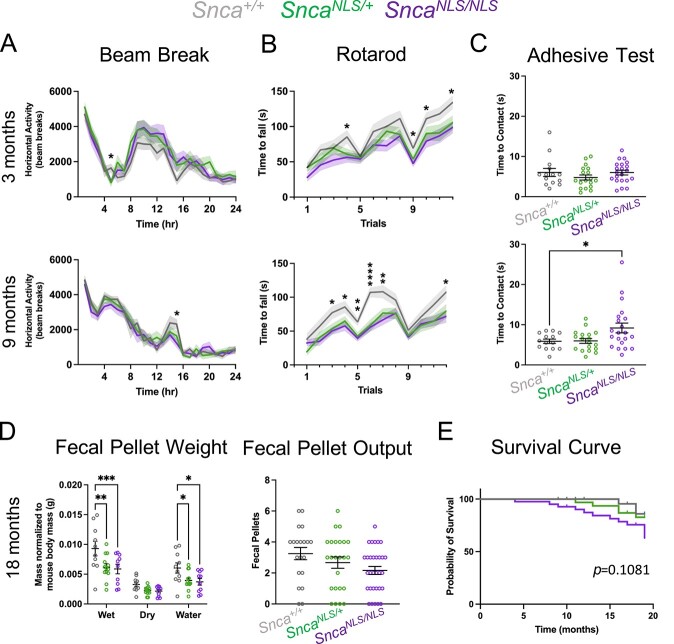
*Snca^NLS^* mice exhibit significant motor and gastrointestinal dysfunction. Analysis of the 3- (top) and 9-month (bottom) mice for (**A**) Beam Break, (**B**) Rotarod and (**C**) Adhesive Test measuring time to contact their forepaws, (**D**) Fecal Pellet Weight measuring fecal weight and water content over 1 h (left), and Fecal Pellet Output measuring fecal pellets produced in 10 min (right), (*n* = 10–21). (**E**) Survival curve from all mice in the behavior colony (*n* = 42–58). One-way (C, D right), two-way ANOVA (A, D left), or mixed-effects analysis (B) with Bonferroni multiple comparison or Log-rank (Mantel Cox) test (**F**): ns, ^*^, ^**^, ^***^ and ^****^ denote *P* > 0.05, < 0.05, < 0.01, < 0.001 and <0.0001, respectively.

To test whether chronic nuclear accumulation of αSyn is sufficient to elicit PD-like phenotypes over time, we subjected *Snca^NLS/NLS^* mice and littermates to a battery of behavior tests at 3-, 9- and 18-months of age. We found that the *Snca^NLS/+^* and *Snca^NLS/NLS^* mice performed similarly to wildtype at 3-months of age (Fig. 3A). By 9-months, however, the *Snca^NLS/NLS^* mice displayed a significant motor deficit in rotarod (Fig. 3B) as well as a delayed time to contact their forepaws in the adhesive test (Fig. 3C). Interestingly, *Snca^NLS/+^* mice also exhibited a significant deficit on the rotarod test—suggestive of a dominant phenotype. This rotarod deficit was not observed when testing *Snca^−/−^* mice at 9 months (Supplementary Material, Fig. S2), suggesting it is due to the accumulation of nuclear αSyn and not a loss-of-function of pre-synaptic αSyn. Surprisingly, after aging the *Snca^NLS^* mice to 18 months, we observed milder motor phenotypes relative to wildtype controls; likely due to the 18-month wildtype mice showing increased difficulty at performing these tasks when comparing their motor ability with that at 3- and 9-months of age (Supplementary Material, Fig. S3).

With increasing awareness around non-motor symptoms in PD, we also measured cognition, anxiety and overall wellness in the *Snca^NLS^* line. We found that *Snca^NLS/NLS^* and *Snca^NLS/+^* mice performed similarly to their wildtype littermates in non-motor behavior tests at all timepoints (Supplementary Material, Figs. S4–S6). In addition to motor decline, people living with PD often experience gastrointestinal difficulties such as constipation (26,27). To measure constipation in our mice, we examined fecal excretions in the span of an hour. We found that 18-month-old *Snca^NLS/NLS^* mice excretions contained significantly less water than their wildtype counterparts (Fig. 3D). Lastly, we observed a trend for early lethality in the *Snca^NLS/NLS^* mice, whereby 37% of *Snca^NLS/NLS^* mice died by 20 months of age, compared with 14% of wildtype littermates (*P* = 0.1081, Fig. 3E). Taken together, the *Snca^NLS/NLS^* mice display age-dependent motor and gastrointestinal dysfunction reminiscent of PD.

### *Snca* ^*NLS/NLS*^ mice exhibit cortical atrophy, independent of αSyn aggregation or dopaminergic neurodegeneration

**Figure 4:**
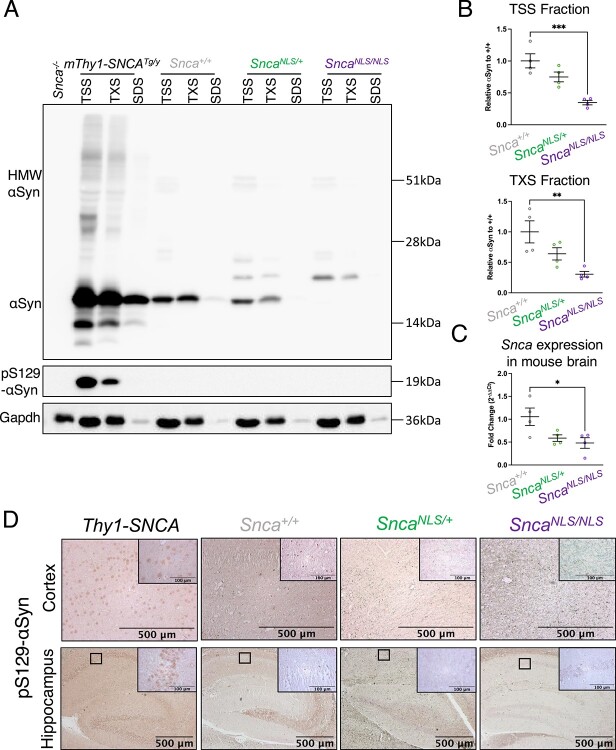
*Snca^NLS/NLS^* mice do not display significant changes in aggregated or phosphorylated αSyn. Serial extraction of cortical mouse brain tissue with western blot probed for (**A**) αSyn (upper blot) and pS129-αSyn (middle blot) and Glyceraldehyde-3-Phosphate Dehydrogenase (Gapdh; bottom blot) comparing the level of αSyn in the (**B**) TSS (upper) and TXS (lower) fraction (*n* = 4). (**C**) qPCR of *Snca* mRNA from 9-month mouse cortex (*n* = 4). (**D**) pS129-αSyn staining of the motor cortex (upper) and hippocampus (lower). One-way ANOVA with Bonferroni multiple comparison: ^*^, ^**^ and ^***^ denotes *P* < 0.05, < 0.01 and <0.001, respectively.

Many studies suggest that αSyn toxicity is intrinsically tied to its aggregation, as the two are often associated in humans with PD and in animal models of the disease (1,28,29). However, models of αSyn toxicity often rely on the introduction of synthetically derived misfolded αSyn fibrils (30,31) or overexpression of αSyn (15,32), thereby potentiating its aggregation *in vivo*. Given that the *Snca^NLS^* mice display age-dependent behavioral phenotypes, yet do not rely on αSyn overexpression, we asked whether accumulation of endogenous αSyn in the nucleus leads to its aggregation and thusly contributes to its toxicity. We examined both the solubility of αSyn as well as its pathologically linked phosphorylation at serine residue 129 (pS129) by biochemical fractionation of brain samples of *Snca^NLS/NLS^* mice compared with littermates. We used the *mThy1-SNCA* (‘line 61’) transgenic and *Snca* knockout (*Snca^−/−^*) mouse lines as positive (32) and negative (33) controls, respectively. To our surprise, we found that the accumulation of nuclear αSyn does not lead to aggregation (Fig. 4A), nor does it become phosphorylated at S129 (Fig. 4A, B), and in fact total αSyn levels are reduced in these mice (Fig. 4A–C). These findings were further supported by histology, which showed no marked increase in pS129 in aged *Snca^NLS/NLS^* mice compared with their respective littermates (Fig. 4D). This suggests that nuclear accumulation of αSyn confers neuronal dysfunction independent of aggregation.

**Figure 5:**
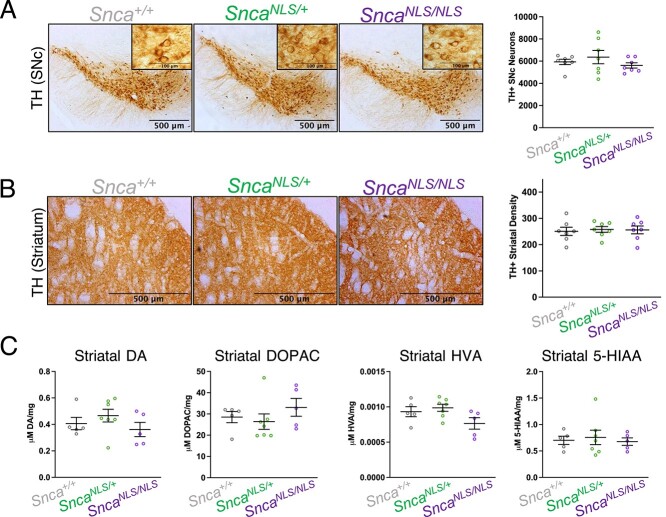
18-month-old *Snca^NLS/NLS^* mice exhibit intact nigrostriatal tracts. Tyrosine hydroxylase staining of the (**A**) Substantia nigra *pars compacta* and (**B**) striatum of 18-month mice (*n* = 7). (**C**) HPLC of 18-month striatal tissue measuring Dopamine (left), DOPAC (middle left), HVA (middle right) and 5-HIAA (right) (*n* = 5–7). One-way ANOVA with Bonferroni multiple comparison: ns denotes *P* > 0.05.

A hallmark of PD is nigrostriatal degeneration. Because of the relatively high expression of αSyn in dopaminergic neurons [Fig. 2C; (34)], we hypothesized nuclear αSyn could be acutely toxic to dopaminergic neurons, causing their death and ultimately leading to the observed behavioral deficits in *Snca^NLS^* mice. To our surprise, we found that young and aged *Snca^NLS/NLS^* mice had intact nigrostriatal tracts, when evaluated by striatal tyrosine hydroxylase (TH) fiber density and stereological estimation of dopaminergic cell number in the SNc at 3- and 18-months of age (Fig. 5A and B, Supplementary Material, Fig. S4E). Moreover, HPLC (high-performance liquid chromatography) analysis of mouse striata revealed that 18-month-old mice across genotypes exhibit similar levels of dopamine and its metabolites (DOPAC, HVA and 5-HIAA; Fig. 5C).

**Figure 6:**
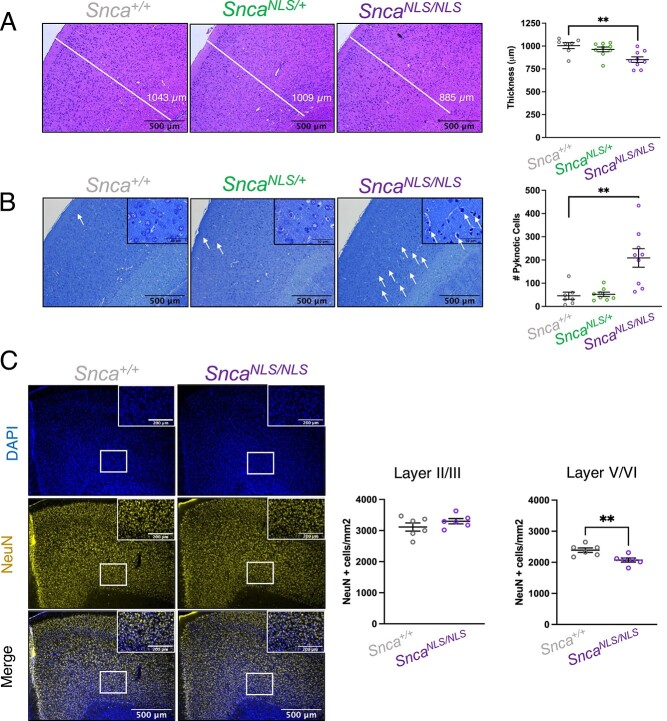
Aged *Snca^NLS/NLS^* mice exhibit cortical atrophy. (**A**) H&E staining with quantification of the motor cortex thickness (*n* = 7–9). (**B**) Toluidine blue staining with quantification of pyknotic cells from the motor cortex (*n* = 7–9). White arrows denote select pyknotic cells. (**C**) NeuN staining of wildtype (left) and *Snca^NLS/NLS^* (right) measuring NeuN^+^ cells in motor cortex layers II/III and V/VI. One-way ANOVA with Bonferroni multiple comparison (A, B) or unpaired, two-tailed *t*-test (C): ns, ^**^ denote *P* > 0.05 and <0.01, respectively.

Since *Snca^NLS/NLS^* mice exhibit motor defects without nigrostriatal degeneration nor αSyn aggregation, we took a step back to ask whether nuclear αSyn may impact other areas of the brain, thus contributing to PD-like phenotypes. Cortical involvement has long been linked to several synucleinopathies including PD, dementia with Lewy bodies (DLB) and PD with dementia [PDD; (29,35–37)]. We therefore explored higher order cortical areas to determine whether *Snca^NLS/NLS^* mice exhibit neurodegenerative features outside of the SNc. We conducted gross anatomical studies using hematoxylin and eosin (H&E) and toluidine blue staining and found significant anterior cortical thinning in the motor cortex (Fig. 6A) and a marked increase in pyknotic cells (Fig. 6B) throughout the cortex of 18-month-old *Snca^NLS/NLS^* mice. We further stained cortical sections with NeuN [Neuronal nuclei antigen, a.k.a. RNA Binding Fox-1 Homolog 3 (Rbfox3)] and found a significant decrease in neurons in layers V/VI of 18-month-old *Snca^NLS/NLS^* mice compared with their littermates (Fig. 6C). Thus, nuclear αSyn causes cortical dysfunction in the absence of its aggregation or nigrostriatal degeneration.

### Unbiased proteomic analysis uncovers reduced Darpp-32, Pde10a and Gng7 levels in *Snca*^*NLS/NLS*^ mice

**Figure 7:**
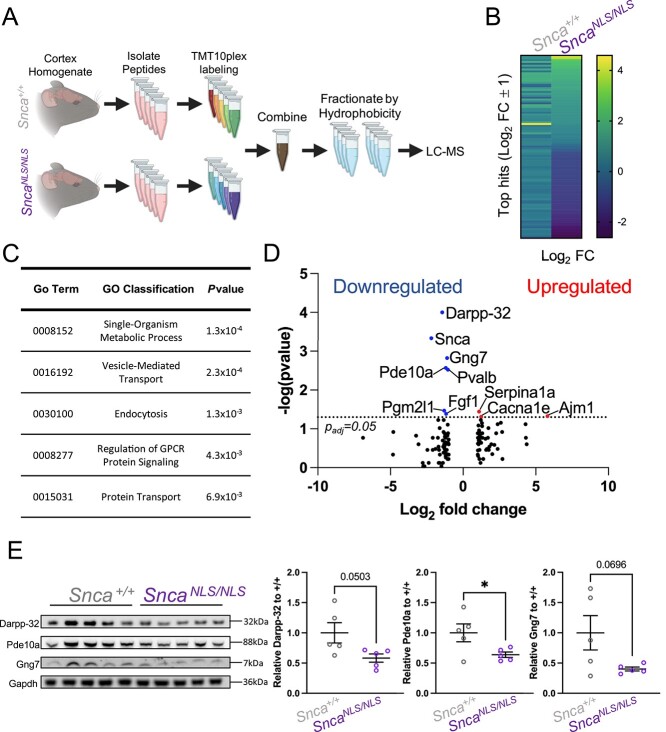
Mass spectrometry reveals proteomic alterations in the *Snca^NLS/NLS^* mice. (**A**) Mass spectrometry scheme for quantitative comparison of proteome between 9-month wildtype and *Snca^NLS/NLS^* cortices (*n* = 5). (**B**) Heat map of all proteins identified through mass spectrometry with values within ±1 Log_2_ fold-change. (**C**) Table of enriched gene ontology pathways among the ±1 Log_2_ fold-change hits. (**D**) Volcano plot of mass spectrometry results with ±1 Log_2_ fold-change highlighting significantly upregulated proteins in red and downregulated in blue. (**E**) Western blot of cortical mouse brain tissue from 9-month mice probing for Darpp-32 (upper), Pde10a (upper middle), Gng7 (lower middle) and Gapdh (bottom) with quantification (right). Unpaired, two-tailed *t*-test: ^*^ denotes *P* < 0.05.

We followed an unbiased strategy to uncover the molecular mechanisms underlying the behavioral and histological phenotypes of the *Snca^NLS/NLS^* mice via quantitative proteomic analysis on cortices from 9-month-old mice. At this age, *Snca^NLS/NLS^* mice exhibit behavioral abnormalities (Fig. 3), allowing identification of early molecular changes that drive the late-stage cortical atrophy exhibited in these mice and in PD. To quantify proteomic differences in wildtype and *Snca^NLS/NLS^* mice, we performed pooled TMT10plex labeling for 5 wildtype and 5 *Snca^NLS/NLS^* mouse cortices followed by mass spectrometry to identify differential proteomic changes (Fig. 7A). This approach yielded a list of nearly 1800 proteins, of which 114 had a Log_2_ fold-change of ±1 relative to wildtype (Fig. 7B). Of these 114 hits, 66 were downregulated and 48 were upregulated (Supplementary Material, Table S2). Gene ontology term analysis revealed a significant enrichment for biological processes that are disturbed in PD, including regulation of GPCR signaling (Fig. 7C). To increase the stringency of our list, we filtered these hits using a statistical cut-off (Mann–Whitney *P*-value < 0.05). From this, we identified 10 high-confidence hits (Fig. 7D). Interestingly, among these 10 hits we noticed a few proteins of particular importance in DA signaling and have been associated with PD, such as Cacna1e, Darpp-32 [dopamine- and cAMP-regulated neuronal phosphoprotein a.k.a. protein phosphatase 1 regulator inhibitor subunit 1b (Ppp1r1b)], Fgf1, Gng7 (G Protein Subunit Gamma 7), Pde10a (Phosphodiesterase 10A) and SerpinA1a (38–45). We confirmed overall trends in reduction of Darpp-32, Pde10a and Gng7 in the 9-month-old *Snca^NLS/NLS^* mice using western blot (Fig. 7E), thereby validating our proteomics approaches. Collectively, these data suggest that disrupted dopaminergic signaling pathways may be an early event of nuclear αSyn toxicity.

## Discussion

The mechanisms underlying αSyn toxicity have been difficult to pin down. We and others have previously shown that nuclear αSyn is increased in the PD post-mortem brain and in animal models harboring *SNCA* mutations (6,13,20,19,46). Nonetheless, previous studies examining the role of nuclear αSyn in PD pathogenesis have yielded conflicting results, ranging from neurodegenerative (13,15,17,46) to neuroprotective (20,25) phenotypes. This may be in part due to the degree of overexpression of αSyn or the choice of read-out in these models. Our study sought to overcome this by answering if the nuclear accumulation of *native* αSyn is sufficient to cause PD-like phenotypes in mice. We engineered a mouse model with an *NLS-Flag* knockin on *Snca* to characterize the effects of chronically increased nuclear αSyn. We found that *Snca^NLS/NLS^* mice reveal PD-like phenotypes including age-dependent motor decline, as well as cortical dysfunction. The cortical atrophy we observed draws parallels to the cell loss seen in synucleinopathies with cortical involvement like DLB and PDD (29,35–37). Moreover, the anatomical location of this cell loss dovetails with the motor deficits seen in these mice and may shed light onto how nuclear αSyn in PD may be linked to cortical dysfunction and disease manifestation. When examining the proteomic profile of the *Snca^NLS/NLS^* mice, we found a few high-confidence hits that have been previously associated with PD. Among these, we identified Pde10a, Darpp-32 and Gng7. Although we found no significant decrease in striatal dopamine, it is interesting to note that these proteins are all involved in dopamine signaling, hinting at a potential mechanism by which nuclear αSyn causes dysfunction via perturbing intracellular signaling (38,42,47–55). Importantly, *Gng7* knockout mice exhibit significant age-dependent motor deficits, particularly in the rotarod test which was the motor assay with which the *Snca^NLS^* mice exhibited the most difficulty (49). In addition, one study looking at patients with schizophrenia found reduced Pde10a and medial prefrontal cortex thinning, another aspect observed in the *Snca^NLS^* mice (55). Together, these changes in protein levels hint at a possible mechanism whereby nuclear αSyn elicits dopaminergic signaling defects, in the absence of overt dopaminergic neurodegeneration.

Although characterizing *Snca^NLS/NLS^* mice, we consistently noted how this mouse line diverges from *Snca^−/−^* mouse phenotypes (Supplementary Material, Fig. S2), cementing that *Snca^NLS/NLS^* mouse phenotypes are likely gain-of-function. To wit, *Snca^−/−^* mice exhibit mild synaptic deficits in the absence of gross motor or non-motor deficits (33,56), likely due to compensation by β-synuclein and, to a lesser extent, γ-synuclein (57–59). Indeed, the motor phenotypes appear to be dependent on the local dose of nuclear αSyn as even the *Snca^NLS/+^* mice exhibit some motor behavior deficits—albeit to a lesser extent than their *Snca^NLS/NLS^* littermates, suggesting that they are due to a gain-of-function of nuclear αSyn and not a loss-of-function of synaptic αSyn. Nevertheless, we cannot exclude a model in which partial loss of synaptic αSyn combined with increased nuclear αSyn may drive the age-dependent behavioural and pathological phenotypes seen in *Snca^NLS^* mice. Strikingly, behavioral and histological phenotypes in *Snca^NLS^* mice occur independently from αSyn aggregation and pathogenic phosphorylation. Indeed, overall levels of αSyn are reduced in *Snca^NLS^* mice—potentially by some regulatory mechanism or feedback loop—therefore suggesting that these αSyn-mediated phenotypes occur despite its aggregation or phosphorylation. This suggests a heretofore underappreciated role of *soluble*, nuclear αSyn in the pathogenesis of PD.

The cellular mechanisms that drive the nuclear accumulation of αSyn and its subsequent sequelae in PD remain elusive. Whether active or passive mechanisms bring αSyn to the nucleus is unknown. Native αSyn does not possess an NLS, therefore, it may be driven into the nucleus by passive mechanisms [it can traverse the nuclear pore complex due to its small size; (60)] and could be retained by interaction with nuclear components (e.g. histones or DNA) (16,17,20,24,25,61) or via uncharacterized modifications. Alternatively, active mechanisms such as its interaction with TRIM28 (19) or RAN (14) may be key in regulating its nuclear import. Moreover, αSyn likely has an important native role in the nucleus, particularly during mouse embryonic development, where nuclear αSyn constitutes up to 40% of its total cellular distribution, compared with 3–15% of total cellular distribution in adult mice (20). There, αSyn is suggested to bind both DNA and histones to modulate gene expression (16,17,24,25,61–64). In wildtype mice, nuclear αSyn was shown to be neuroprotective by binding to DNA and colocalizing with DNA damage response elements to protect against DNA damage (25). Whether the increase in nuclear αSyn observed in PD—modeled in the *Snca^NLS^* mice—causes a gain of this normal developmental function or a neomorphic function will be important to establish, to facilitate future therapeutic development.

## Materials and Methods

### Mouse design and engineering

#### Mouse engineering

To generate the *Snca^NLS^* mice, Cas9 protein was complexed to a sgRNA targeting the 3′ locus of *Snca* (sgRNA target sequence: 5′-TTGGTAGCCTTCCTAATATC-3′); and, together with a single-stranded oligodeoxynucleotide (ssODN) repair template (sequence: 5′-CACTGTGAAGCAGACAGTTGATATCTGTCACTTCACTGACAAGGCATGCTGTTATTATTTTCTTTTTCTGATATTAGGAAGGCTACCAAGACTATGAGCCTGAAGCCGACTACAAGGACGACGACGACCAAGTAAGAATGTCATTGCACCCAATCTCCTAAGATCTGCCGGCTGCTCTTCCATGGCGTACAAGTGCTCAGT-3′; IDT Ultramer), were injected and implanted into pseudo-pregnant FVB female mice (65). Three founder mice were generated and backcrossed onto a ~99.75% pure C57Bl6/J background (Taconic 1450 SNP analysis, sequencing and subsequent backcrossing; see Supplementary Material, Fig. S1A and B), before being expanded onto a mixed C57Bl6/J; C57Bl6/NCrl background. One line was selected for subsequent extensive characterization and is being made available through the Jackson Laboratory (RRID:IMSR_JAX:036763).

#### Genotyping

A small (~1 mm) tail sample is digested prior to PCR amplification using primers outside of the sequence covered by the ssODN used for the initial mouse line. Forward: 5′-TTTTATCTGATTGAAATGATGAGC-3′; Reverse: 5′-ATGACTGGGCACATTGGAA-3′. PCR protocol: 95°C for 2 min, (95°C for 30 s, 56°C for 30 s, 72°C for 30 s) repeated for 35 cycles, 72°C for 5 min. Mutant allele: 273 bp; Wildtype allele: 225 bp.

### Mouse husbandry

All mice were housed with up to 5 mice per cage on a 12 h light–dark cycle. Mice were fed *ad libitum* and all husbandry was performed by the uOttawa Animal Care and Veterinary Services staff. All animal work was done under the approved breeding (CMMb-3009 and CMMb-3654) and behavior (CMMe-3091) protocols approved under the uOttawa Animal Care Committee. All mice were handled daily for 1 week prior to all behavior testing and both male and female mice were used in all experiments.

### Behavior

#### Open field

Lighting in the behavior room was set to 100 lux and mice were habituated for 60 min prior to testing. Mice were placed into the open field box (45 cm^3^) for 10 min with their movement recorded/analyzed with Ethovision software (Noldus Information Technology). dx.doi.org/10.17504/protocols.io.b5qzq5x6.

#### Fecal pellet output

Upon completion of the Open Field test, the number of fecal pellets excreted during the 10 min trial were quantified.

#### Beam break

Single mice were placed into a clean cage with access to food and water *ad libitum* for 24 h at the standard 12 h light–dark cycle with their movement recorded/analyzed via Fusion software.

*Nesting*: Directly following Beam Break testing, one square of nestlet (5 cm^2^ cotton pad) was placed in each Beam Break cage for 17–19 h. Following this, the nestlets were scored on a scale of 1–5 as described in Deacon 2006 (66), with 1 and 5 representing minimal and maximal nest quality, respectively. dx.doi.org/10.17504/protocols.io.b5q2q5ye.

#### Y maze forced alternation

Mice were provided with extra-maze (irregular black cue with squared edges on right wall, black triangle on left wall) and intra-maze (Arm 1 has solid black rectangle, Arm 2 has horizontal bars and Arm 3 has diagonal stripes) cues. The room was set to 60 lux and mice were habituated for 60 min prior to testing. During the first 5 min trial, Arms 2 and 3 were alternately blocked. Following the 30 min inter-trial interval, the mice were placed back into the Y maze apparatus for 5 min without any blocked arms and their movements were recorded/analyzed with EthoVision software. dx.doi.org/10.17504/protocols.io.b5smq6c6.

#### Adhesive test

After a 60 min habituation, the home cage of the mice was lightly wiped to remove all bedding material. The mice were individually placed back into the emptied home cage for a 1 min habituation. Next, a 1 cm^2^ of medical adhesive was placed on each forepaw and the mice were placed back into the wiped home cage where the time to remove adhesive was measured up to a 2 min maximum. dx.doi.org/10.17504/protocols.io.b5snq6de.

#### Pole test

Following a 60 min habituation, mice were placed on a textured metal pole (8 mm diameter, 55 cm tall) ~3 cm from the top facing upwards. The mice were given up to 1 min to turn around (facing downwards) and up to 1 min to descend the pole. dx.doi.org/10.17504/protocols.io.b5sqq6dw.

#### Rotarod

Following a 60 min habituation, mice were placed on a rod (IITC Life Sciences) rotating from 4 to 40 revolutions per minute over 5 min for 4 trials per day with a 10 min inter-trial interval. This was repeated for 3 days total. dx.doi.org/10.17504/protocols.io.b5srq6d6.

#### DigiGait

Following a 60 min habituation, mice were placed in the DigiGait treadmill (Mouse Specifics Inc.). The treadmill ran at 22 cm/s (3- and 9-months timepoints) or 18 cm/s (18-month timepoint) with a 0° incline. 3 s of continuous movement was recorded using DigiGait Imager software and was then analyzed with DigiGait Analysis software. dx.doi.org/10.17504/protocols.io.b5stq6en.

#### Fear conditioning

Naïve age- and sex-matched mice were used to obtain the optimal intensity of foot shock. On Day 1 of testing, mice were placed into the Fear Conditioning apparatus (Noldus Information Technology) for 6 min during which time the mice experienced 3 tone-shock pairings (30 s tone co-terminated with a 2 s foot shock). On Day 2, mice were placed into the same Fear Conditioning apparatus for 6 min with no tone or foot shock. On Day 3, mice were placed into a different Fear Conditioning apparatus, now altered into a triangular shape with solid floor and a vanilla scent for 3 min with no tone then 3 min with the same 30 s tone but no foot shock. Freezing was measured/analyzed with EthoVision on all 3 testing days. dx.doi.org/10.17504/protocols.io.b5suq6ew.

#### Fecal pellet composition

Mice were placed in a clean cage and their fecal pellets were collected over a 1 h period. These pellets were weighed (wet weight) then desiccated at 65°C for 19 h and reweighed (dry weight). The differences were calculated between these values to determine the water content. dx.doi.org/10.17504/protocols.io.b5svq6e6.

### Histology

See Supplementary Material, Table S1 for a comprehensive list of antibodies used in this study.

#### Perfusion

Mice were sedated with 120 mg/kg Euthanyl (DIN00141704) then perfused with 1X phosphate buffered saline (PBS) then 4% paraformaldehyde (PFA). Brains were then extracted and incubated in 4% PFA for 72 h prior to a 3-step sucrose dehydration with 10, 20 and 30% sucrose (24 h each). Next, brains were flash frozen for 1 min in −40°C isopentane and cryosectioned at 40 μm. dx.doi.org/10.17504/protocols.io.b5swq6fe.

#### Immunofluorescent staining

Cryosectioned tissue was mounted on a slide then blocked with blocking buffer (0.5% Triton X-100, 10% normal horse serum in 1X PBS) then incubated in primary antibody overnight at 4°C. Next, the sections were incubated in secondary antibody before drying at room temperature for 2 min. The sections were then covered with #1.5 coverslips and fluorescent mounting medium (Dako cat# S3023). dx.doi.org/10.17504/protocols.io.b5s5q6g6.

#### Quick decapitation, fixation

Mice were euthanized by isoflurane inhalation followed by decapitation and brains were quickly extracted. Brains were then submerged in 10% buffered formalin for 72 h prior to paraffin embedding and sectioning at 5 μm. dx.doi.org/10.17504/protocols.io.b5szq6f6.

#### Diaminobenzidine (DAB) staining

Paraffin-embedded sections were deparaffinized in serial baths of xylenes and ethanol prior to a sodium citrate (10 mm sodium citrate, 0.05% Tween-20, pH 6) antigen retrieval (20 min at 95°C) and 0.9% H_2_O_2_ treatment (10 min). Next, sections were blocked in blocking buffer (0.1% Triton X-100, 10% normal horse serum in 1X PBS) then incubated in primary antibody overnight at 4°C. The following day the sections were incubated in secondary and tertiary antibody solution before exposure to DAB, dehydrating in baths of ethanol and xylenes and covering the tissue with Permount (Fisher Scientific cat# SP15–100) and #1.5 coverslips. dx.doi.org/10.17504/protocols.io.b5s9q6h6.

#### Toluidine Blue and H&E staining

Staining was performed by the Louise Pelletier Histology Core facility at the University of Ottawa on paraffin-embedded 5 μm sectioned mouse brain tissue using the Leica Autostainer XL. Briefly, the sections were deparaffinized and exposed to Toluidine Blue for 10 min. For H&E, sections were deparaffinized, exposed to hematoxylin for 7 min and eosin for 30 s. Then, sections were dehydrated and covered with #1.5 coverslips. Two serial sections for each were analyzed by an investigator blinded to the genotypes.

#### Stereology

Stereology was performed as previously described (19). Briefly, for each mouse, 8 cryosections were stained for TH and quantified using StereoInvestigator software (version 11.06.2). The sections (40 μm) began at the outer limit of the substantia nigra (SNc) and every 6th section was used (Bregma −2.54 to −3.88 mm). Mean section thickness was determined during counting at a frequency of 10 frames (roughly three measurements per hemisphere). The SNc was sampled by randomly translating a grid with 150 μm × 150 μm squares in the outlined SNc and applying an optical fractionator consisting of a 75 μm × 75 μm square. All stereological analyses were performed by an investigator blinded to genotypes dx.doi.org/10.17504/protocols.io.b5ynq7ve.

#### TH densitometry

For each mouse, 4 cryosections were stained for TH and imaged with Zeiss Axio Imager M2 and analyzed in ImageJ (version 2.3.0/1.53f). The sections (40 μm) began at the outer limit of the striatum and every 12th section was used (Bregma 1.18 to −0.22 mm). Brightfield images were captured at 10X magnification and converted to greyscale. To analyze, an investigator blinded to genotypes averaged 20 intensity measurements within the dorsolateral striatum and normalized to the average of 5 intensity measurements in the corpus callosum (background staining control). All 4 cryosections per mouse were averaged and represent 1 datapoint on the graph dx.doi.org/10.17504/protocols.io.b5u4q6yw.

#### Primary cortical neurons

Micro Coverglass #1.5 (Electron Microscopy Sciences) coverslips were pre-coated with poly-d-lysine (50 μg/ml) for at least overnight at 37°C, then washed with distilled water twice and air dried at room temperatue for 2 h. Primary neurons were harvested from E13.5 to 15.5 pups and seeded at ~1 × 10^5^ cells per coverslip and cultured for 7 days *in vitro*. Cells were fixed in 4% PFA for 20 min, washed in 1X PBS, then blocked (10% serum +0.1% Triton X-100) for 1 h. Coverslips were stained overnight (see above immunofluorescent staining method) dx.doi.org/10.17504/protocols.io.b5u6q6ze.

#### Immunofluorescent imaging

Images for Figures 1 and 2 were obtained on a Zeiss AxioObserverZ1 LSM800 Confocal Microscope. Primary neuron images (Fig. 1) were obtained at 40X (oil) magnification at 8bit 1024 × 1024 resolution through a *Z* distance of ~10 μm per image using optimal 0.27 μm spacing. Multichannel acquisition was performed using excitation/emissions: AF488 (Syn1) 493/517 nm (710V); DAPI 353/465 nm (750V); AF647 (Map2) 653/668 nm (865V) and AF594 (αSyn) 280/618 nm (700V). Images from tissue sections (Fig. 2) were obtained at 20X magnification (0.8× zoom) at 16bit 1024 × 1024 resolution through a range of 10 μm per image using optimal 0.5 μm spacing. Multichannel acquisition was performed using excitation/emissions (laser intensity); AF488 (αSyn) 493/517 nm (630V for Cortex and DG, 620V for SNc); DAPI 353/465 nm (650V for Cortex and DG, 630V for SNc); AF647 (TH) 653/668 nm (600V). All images were processed and analyzed in ImageJ2 (version 2.3.0/1.53f) using the 3D project and Maximum Z project functions for primary neuron and tissue sections, respectively. Images for Figure 6C were obtained on a Zeiss Axio Imager M2 at 5X magnification and were analyzed in ImageJ2 (version 2.3.0/1.53f).

#### Nuclear αSyn quantification

Primary cortical neurons were imaged using confocal imaging and all cells in the frame of view were used to quantify the extent of aSyn nuclear localization using ImageJ2 (version 2.3.0/1.53f). First, the nuclei were delineated with DAPI then aSyn intensity was measured within the delineation. This data is shown as the level of aSyn nuclear enrichment relative to wildtype littermates. Cortex, DG and SNc were analyzed in a similar manner. Briefly, 11–35 cells were sampled per animal using ImageJ2 (version 2.3.0/1.53f), nuclei were defined with DAPI and relative intensity of nuclear αSyn versus cytoplasmic αSyn (immediately adjacent to the nucleus) was measured.

#### NeuN analysis

Photomicrographs were analyzed using ImageJ2 (version 2.3.0/1.53f). Briefly, NeuN positive structures (neuronal nuclei) were counted in a 200 μM × 200 μM frame (Layer II/III) or 350 μM × 350 μM frame (Layer V/VI) and then presented as NeuN+ neuron #/mm^2^. Values represent the average from sampling three distinct sections per brain (*n* = 6 per genotype) and were analyzed by an investigator blinded to the genotypes.

### Biochemistry

See Supplementary Material, Table S1 for a comprehensive list of antibodies used in this study.

#### Serial extraction

A 0.79 mm^3^ punch (1 mm thick slice, 1 mm i.d. punch) of cortical brain tissue from 18-month-old mice was homogenized and resuspended in a series of increasingly stringent buffers beginning with 100 μl of TSS Buffer (140 mm NaCl, 5 mm Tris–HCl), then 100 μl TXS Buffer (140 mm NaCl, 5 mm Tris–HCl, 0.5% Triton X-100), then 100 μl SDS Buffer (140 mm NaCl, 5 mm Tris–HCl, 1% SDS), as previously described (67). Total protein levels were measured using the Pierce™ BCA Assay Kit (Thermo Fisher cat# 23225). dx.doi.org/10.17504/protocols.io.b5vvq666.

#### Western blot

Protein samples were loaded into a 12% polyacrylamide gel and subsequently transferred to a 0.2 μm nitrocellulose membrane. Membranes were blocked in a 10% milk solution then incubated in primary antibody (diluted in 2% bovine serum albumin) overnight at 4°C. Next, the membrane was incubated in a horseradish peroxidase-conjugated secondary antibody diluted in 10% milk solution. Then, the membrane was rinsed with ECL Clarity solution (Bio-Rad cat# 1705061) and imaged with a GE ImageQuant LAS 4000. dx.doi.org/10.17504/protocols.io.b5wkq7cw.

#### RNA extraction and real-time quantitative PCR

RNA was extracted from mouse brain homogenate using Trizol-Chloroform extraction (Invitrogen™ User Guide: TRIzol Reagent version B.0). Briefly, mouse brains were homogenized in 3 ml of PEPI Buffer [5 mm EDTA, 1X protease inhibitor (GenDEPOT cat# P3100-020), in 1X PBS] using a dounce homogenizer. 3% of homogenate was added to 1 ml of TRIzol Reagent (Fisher Scientific cat# 15-596-026) and RNA was isolated following the user guide referenced above. cDNA was synthesized using 5X All-in-One RT Master Mix Kit (Bio Basic cat# HRT025-10). Real-time quantitative PCR was performed using Green-2-Go qPCR Master Mix (Bio Basic cat# QPCR004-S) with 25 ng cDNA per reaction and primers targeting mouse *Gapdh* (Forward: 5′-GGAGAGTGTTTCCTCGTCCC-3′, Reverse: 5′-ATGAAGGGGTCGTTGATGGC-3′), *Hprt1* (Forward: 5′- TGATAGATCCATTCCTATGACTGTAGA-3′, Reverse: 5′-AAGACATTCTTTCCAGTTAAAGTTGAG-3′), and *Snca* (Forward: 5′-GAAGACAGTGGAGGGAGCTG-3′, Reverse: 5′-CAGGCATGTCTTCCAGGATT-3′). Reactions were run on BioRad CFX96 thermocycler (protocol: 95°C for 5 min, 40 cycles of 95°C for 15 s and 60°C for 60 s, then melting curve). *Snca* Ct values were standardized to the average of *Hprt1* and *Gapdh*  dx.doi.org/10.17504/protocols.io.b5wmq7c6.

#### Dopamine and metabolite measurement via liquid chromatography-mass spectrometry/mass spectrometry (LC–MS/MS)

Striatal punches (2 mm i.d., 3 mm thick section) were extracted from 18-month-old mouse brains and weighed prior to submitting to The Metabolomics Innovation Centre (TMIC). Fifty microliter of tissue extraction buffer was added to each sample tube followed by homogenization and centrifugation. Supernatant was used for LC–MS/MS analysis to get concentrations in the unit of μM. TMIC staff applied a targeted quantitative metabolomics approach to analyze the samples using a reverse-phase LC–MS/MS custom assay. This custom assay, in combination with an ABSciex 4000 QTrap (Applied Biosystems/MDS Sciex) mass spectrometer, can be used for the targeted identification and quantification of dopamine (DA), homovanillic acid (HVA), 5-hydroxyindoleacetic acid (5-HIAA) and 3,4-dihydroxyphenylacetic acid (DOPAC). The method combines the derivatization and extraction of analytes, and the selective mass-spectrometric detection using multiple reaction monitoring (MRM) pairs. Isotope-labeled internal standards and other internal standards are used for metabolite quantification. The custom assay contains a 96 deep-well plate with a filter plate attached with sealing tape, and reagents and solvents used to prepare the plate assay. First 14 wells were used for 1 blank, 3 zero samples, 7 standards and 3 quality control samples. For all metabolites except organic acid, samples were thawed on ice and were vortexed and centrifuged at 13 000×*g*. Ten microliter of each sample was loaded onto the center of the filter on the upper 96-well plate and dried in a stream of nitrogen. Subsequently, phenyl-isothiocyanate was added for derivatization. After incubation, the filter spots were dried again using an evaporator. Extraction of the metabolites was then achieved by adding 300 μl of extraction solvent. The extracts were obtained by centrifugation into the lower 96-deep well plate, followed by a dilution step with MS running solvent.

For organic acid analysis, 150 μl of ice-cold methanol and 10 μl of isotope-labeled internal standard mixture was added to 50 μl of sample for overnight protein precipitation. Then it was centrifuged at 13 000×*g* for 20 min. Fifty microliter of supernatant was loaded into the center of the wells of a 96-deep well plate, followed by the addition of 3-nitrophenylhydrazine (NPH) reagent. After incubation for 2 h, BHT stabilizer and water were added before LC–MS injection.

Mass spectrometric analysis was performed on an ABSciex 4000 Qtrap® tandem mass spectrometry instrument (Applied Biosystems/MDS Analytical Technologies, Foster City, CA) equipped with an Agilent 1260 series UHPLC system (Agilent Technologies, Palo Alto, CA). The samples were delivered to the mass spectrometer by a LC method followed by a direct injection (DI) method. Data analysis was done using Analyst 1.6.2.

#### TMT10plex™ proteomics via liquid chromatography-mass spectrometry

Whole mouse cortex was dissected from 9-month-old mouse brain and peptides were isolated using the EasyPep™ Mini MS Sample Prep Kit (Thermofisher cat# A40006) following manufacturer instructions. These samples were labelled with the TMT10plex™ Isobaric Label Reagent Set (Thermofisher cat# 90406) then combined into a single tube and fractionated into 12 samples using the Pierce™ High pH Reversed-Phase Peptide Fractionation Kit (Thermofisher cat# 84868). Fractions 2, 3, 9, 10, 11 and 12 were combined due to low protein concentration (combined to have a consistent protein concentration with other fractions) and the 6 final fractions were submitted for liquid chromatography-mass spectrometry (LC–MS) to the Ottawa Hospital Research Institute Proteomics Core Facility. LC–MS was performed using Orbitrap Fusion Lumos mass spectrometer with UltiMate 3000 RLSC nano HPLC (Thermo Scientific). Proteowizard MS-CONVERT was used to generate peak lists for preliminary qualitative analysis using MASCOT software version 2.7.0 (Matrix Science, UK). Protein identification and quantitative analysis was performed using MaxQuant (Tyanova, Nature Protocols 2016, 11:2301). The reference proteome for peptide spectrum matching was UniProt/*Mus musculus* (version 2020-10-06). The MaxQuant results were exported to Scaffold Q + S (Proteome Software, USA) for further analysis and viewing. The mass spectrometry proteomics data have been deposited to the ProteomeXchange Consortium via the PRIDE (68) partner repository with the dataset identifier PXD032065 and http://proteomecentral.proteomexchange.org/cgi/GetDataset?ID=PXD032065}{10.6019/PXD032065}.

### Statistical analyses

All statistical analyses were performed using GraphPad Prism (version 9.1.2) using the appropriate statistical test, either Student’s *t*-test for simple comparisons or one- or two-way analysis of variance (ANOVA) followed by Bonferroni post-hoc analysis for multiple comparisons. The survival curve was analyzed using a log-rank Mantel–Cox test. The Mann–Whitney test with Benjamini–Hochberg correction was used with Scaffold (version 5.0.0) to analyze the TMT10plex™ mass spectrometry dataset. Chi squared test was used to compare actual and expected Mendelian ratios of the genotypes. Statistical tests used, sample sizes, and *P*-values are delineated in each figure legend. All graphs plot the mean with the standard error of the mean (SEM). All figures and their quantification have been made public (https://zenodo.org/record/6082270\#.YkuVtNtBzIU}{doi.org/10.5281/zenodo.6082270}).

## Supplementary Material

Supplementary_Table_1_ddac035

Supplementary_Table_2_ddac035

Supplementary_Figure_1_ddac035

Supplementary_Figure_2_ddac035

Supplementary_Figure_3_ddac035

Supplementary_Figure_4_ddac035

Supplementary_Figure_5_ddac035

Supplementary_Figure_6_ddac035

supplementary_legends_ddac035

Slide8_ddac035

Slide9_ddac035

Slide10_ddac035

Slide11_ddac035

Slide13_ddac035

## References

[1] Spillantini, M.G., Schmidt, M.L., Lee, V.M., Trojanowski, J.Q., Jakes, R. and Goedert, M. (1997) Alpha-synuclein in Lewy bodies. Nature, 388, 839–840. 9278044 10.1038/42166

[2] Polymeropoulos, M.H., Lavedan, C., Leroy, E., Ide, S.E., Dehejia, A., Dutra, A., Pike, B., Root, H., Rubenstein, J., Boyer, R. et al. (1997) Mutation in the alpha-synuclein gene identified in families with Parkinson’s disease. Science, 276, 2045–2047. 9197268 10.1126/science.276.5321.2045

[3] Krüger, R., Kuhn, W., Müller, T., Woitalla, D., Graeber, M., Kösel, S., Przuntek, H., Epplen, J.T., Schöls, L. and Riess, O. (1998) Ala30Pro mutation in the gene encoding alpha-synuclein in Parkinson’s disease. Nat. Genet., 18, 106–108. 9462735 10.1038/ng0298-106

[4] Zarranz, J.J., Alegre, J., Gómez-Esteban, J.C., Lezcano, E., Ros, R., Ampuero, I., Vidal, L., Hoenicka, J., Rodriguez, O., Atarés, B. et al. (2004) The new mutation, E46K, of α-synuclein causes parkinson and Lewy body dementia. Ann. Neurol., 55, 164–173. 14755719 10.1002/ana.10795

[5] Appel-Cresswell, S., Vilarino-Guell, C., Encarnacion, M., Sherman, H., Yu, I., Shah, B., Weir, D., Thompson, C., Szu-Tu, C., Trinh, J. et al. (2013) Alpha-synuclein p.H50Q, a novel pathogenic mutation for Parkinson’s disease. Mov. Disord. Off. J. Mov. Disord. Soc., 28, 811–813. 10.1002/mds.25421 23457019

[6] Fares, M.-B., Ait-Bouziad, N., Dikiy, I., Mbefo, M.K., Jovičić, A., Kiely, A., Holton, J.L., Lee, S.-J., Gitler, A.D., Eliezer, D. et al. (2014) The novel Parkinson’s disease linked mutation G51D attenuates in vitro aggregation and membrane binding of α-synuclein, and enhances its secretion and nuclear localization in cells. Hum. Mol. Genet., 23, 4491–4509. 24728187 10.1093/hmg/ddu165 PMC4119404

[7] Ghosh, D., Sahay, S., Ranjan, P., Salot, S., Mohite, G.M., Singh, P.K., Dwivedi, S., Carvalho, E., Banerjee, R., Kumar, A. et al. (2014) The newly discovered Parkinson’s disease associated finnish mutation (A53E) attenuates α-synuclein aggregation and membrane binding. Biochemistry, 53, 6419–6421. 25268550 10.1021/bi5010365

[8] Konno, T., Ross, O.A., Puschmann, A., Dickson, D.W. and Wszolek, Z.K. (2016) Autosomal dominant Parkinson’s disease caused by SNCA duplications. Parkinsonism Relat. Disord., 22(Suppl 1), S1–S6. 26350119 10.1016/j.parkreldis.2015.09.007 PMC4820832

[9] Maroteaux, L., Campanelli, J.T. and Scheller, R.H. (1988) Synuclein: a neuron-specific protein localized to the nucleus and presynaptic nerve terminal. J. Neurosci., 8, 2804–2815. 3411354 10.1523/JNEUROSCI.08-08-02804.1988 PMC6569395

[10] Herman, S.A. and Coffin, J.M. (1986) Differential transcription from the long terminal repeats of integrated avian leukosis virus DNA. J. Virol., 60, 497–505. 3021984 10.1128/jvi.60.2.497-505.1986 PMC288918

[11] Kahle, P.J., Neumann, M., Ozmen, L., Muller, V., Jacobsen, H., Schindzielorz, A., Okochi, M., Leimer, U., van Der Putten, H., Probst, A. et al. (2000) Subcellular localization of wild-type and Parkinson’s disease-associated mutant alpha -synuclein in human and transgenic mouse brain. J. Neurosci., 20, 6365–6373. 10964942 10.1523/JNEUROSCI.20-17-06365.2000 PMC6772969

[12] Specht, C.G., Tigaret, C.M., Rast, G.F., Thalhammer, A., Rudhard, Y. and Schoepfer, R. (2005) Subcellular localisation of recombinant alpha- and gamma-synuclein. Mol. Cell. Neurosci., 28, 326–334. 15691713 10.1016/j.mcn.2004.09.017

[13] Jiang, P., Gan, M., Yen, S.-H., Moussaud, S., McLean, P.J. and Dickson, D.W. (2016) Proaggregant nuclear factor(s) trigger rapid formation of α-synuclein aggregates in apoptotic neurons. Acta Neuropathol. (Berl.), 132, 77–91. 26839082 10.1007/s00401-016-1542-4 PMC4911378

[14] Chen, V., Moncalvo, M., Tringali, D., Tagliafierro, L., Shriskanda, A., Ilich, E., Dong, W., Kantor, B. and Chiba-Falek, O. (2020) The mechanistic role of alpha-synuclein in the nucleus: impaired nuclear function caused by familial Parkinson’s disease SNCA mutations. Hum. Mol. Genet., 29, 3107–3121. 32954426 10.1093/hmg/ddaa183 PMC7645704

[15] Masliah, E., Rockenstein, E., Veinbergs, I., Mallory, M., Hashimoto, M., Takeda, A., Sagara, Y., Sisk, A. and Mucke, L. (2000) Dopaminergic loss and inclusion body formation in α-synuclein mice: implications for neurodegenerative disorders. Science, 287, 1265–1269. 10678833 10.1126/science.287.5456.1265

[16] Goers, J., Manning-Bog, A.B., McCormack, A.L., Millett, I.S., Doniach, S., Di Monte, D.A., Uversky, V.N. and Fink, A.L. (2003) Nuclear localization of alpha-synuclein and its interaction with histones. Biochemistry, 42, 8465–8471. 12859192 10.1021/bi0341152

[17] Kontopoulos, E., Parvin, J.D. and Feany, M.B. (2006) Alpha-synuclein acts in the nucleus to inhibit histone acetylation and promote neurotoxicity. Hum. Mol. Genet., 15, 3012–3023. 16959795 10.1093/hmg/ddl243

[18] Huang, Z., Xu, Z., Wu, Y. and Zhou, Y. (2011) Determining nuclear localization of alpha-synuclein in mouse brains. Neuroscience, 199, 318–332. 22033456 10.1016/j.neuroscience.2011.10.016 PMC3237852

[19] Rousseaux, M.W., de Haro, M., Lasagna-Reeves, C.A., De Maio, A., Park, J., Jafar-Nejad, P., Al-Ramahi, I., Sharma, A., See, L., Lu, N. et al. (2016) TRIM28 regulates the nuclear accumulation and toxicity of both alpha-synuclein and tau. elife, 5, e19809. 27779468 10.7554/eLife.19809 PMC5104516

[20] Pinho, R., Paiva, I., Jercic, K.G., Fonseca-Ornelas, L., Gerhardt, E., Fahlbusch, C., Garcia-Esparcia, P., Kerimoglu, C., Pavlou, M.A.S., Villar-Piqué, A. et al. (2019) Nuclear localization and phosphorylation modulate pathological effects of alpha-synuclein. Hum. Mol. Genet., 28, 31–50. 30219847 10.1093/hmg/ddy326

[21] Koss, D.J., Erskine, D., Porter, A., Leite, M., Attems, J. and Outeiro, T.F. (2021) Alpha-synuclein is present in the nucleus in human brain tissue and is pathologically modified in dementia with Lewy bodies. BioRxiv, 2021.10.20.465125. 10.1186/s40478-022-01403-x PMC9258129 35794636

[22] McLean, P.J., Ribich, S. and Hyman, B.T. (2000) Subcellular localization of alpha-synuclein in primary neuronal cultures: effect of missense mutations. J. Neural Transm. Suppl., 53–63. 10.1007/978-3-7091-6284-2_5 11128613

[23] Ciron, C., Zheng, L., Bobela, W., Knott, G.W., Leone, T.C., Kelly, D.P. and Schneider, B.L. (2015) PGC-1α activity in nigral dopamine neurons determines vulnerability to α-synuclein. Acta Neuropathol. Commun., 3, 16. 25853296 10.1186/s40478-015-0200-8 PMC4379693

[24] Paiva, I., Jain, G., Lázaro, D.F., Jerčić, K.G., Hentrich, T., Kerimoglu, C., Pinho, R., Szegő, È.M., Burkhardt, S., Capece, V. et al. (2018) Alpha-synuclein deregulates the expression of COL4A2 and impairs ER-Golgi function. Neurobiol. Dis., 119, 121–135. 30092270 10.1016/j.nbd.2018.08.001

[25] Schaser, A.J., Osterberg, V.R., Dent, S.E., Stackhouse, T.L., Wakeham, C.M., Boutros, S.W., Weston, L.J., Owen, N., Weissman, T.A., Luna, E. et al. (2019) Alpha-synuclein is a DNA binding protein that modulates DNA repair with implications for Lewy body disorders. Sci. Rep., 9, 1–19. 31358782 10.1038/s41598-019-47227-z PMC6662836

[26] Siddiqui, M.F., Rast, S., Lynn, M.J., Auchus, A.P. and Pfeiffer, R.F. (2002) Autonomic dysfunction in Parkinson’s disease: a comprehensive symptom survey. Parkinsonism Relat. Disord., 8, 277–284. 12039423 10.1016/s1353-8020(01)00052-9

[27] Cheon, S.-M., Ha, M.-S., Park, M.J. and Kim, J.W. (2008) Nonmotor symptoms of Parkinson’s disease: prevalence and awareness of patients and families. Parkinsonism Relat. Disord., 14, 286–290. 18042421 10.1016/j.parkreldis.2007.09.002

[28] Baba, M., Nakajo, S., Tu, P.H., Tomita, T., Nakaya, K., Lee, V.M., Trojanowski, J.Q. and Iwatsubo, T. (1998) Aggregation of alpha-synuclein in Lewy bodies of sporadic Parkinson’s disease and dementia with Lewy bodies. Am. J. Pathol., 152, 879–884. 9546347 PMC1858234

[29] Braak, H., Tredici, K.D., Rüb, U., de Vos, R.A.I., Jansen Steur, E.N.H. and Braak, E. (2003) Staging of brain pathology related to sporadic Parkinson’s disease. Neurobiol. Aging, 24, 197–211. 12498954 10.1016/s0197-4580(02)00065-9

[30] Luk, K.C., Kehm, V.M., Zhang, B., O’Brien, P., Trojanowski, J.Q. and Lee, V.M.Y. (2012) Intracerebral inoculation of pathological α-synuclein initiates a rapidly progressive neurodegenerative α-synucleinopathy in mice. J. Exp. Med., 209, 975–986. 22508839 10.1084/jem.20112457 PMC3348112

[31] Luk, K.C., Kehm, V., Carroll, J., Zhang, B., O’Brien, P., Trojanowski, J.Q. and Lee, V.M.-Y. (2012) Pathological α-synuclein transmission initiates Parkinson-like neurodegeneration in non-transgenic mice. Science, 338, 949–953. 23161999 10.1126/science.1227157 PMC3552321

[32] Chesselet , Richter, F., Zhu, C., Magen, I., Watson, M.B. and Subramaniam, S.R. (2012) A progressive mouse model of Parkinson’s disease: the Thy1-aSyn (“line 61”) mice. Neurotherapeutics, 9, 297–314. 22350713 10.1007/s13311-012-0104-2 PMC3337020

[33] Cabin, D.E., Shimazu, K., Murphy, D., Cole, N.B., Gottschalk, W., McIlwain, K.L., Orrison, B., Chen, A., Ellis, C.E., Paylor, R. et al. (2002) Synaptic vesicle depletion correlates with attenuated synaptic responses to prolonged repetitive stimulation in mice lacking alpha-synuclein. J. Neurosci., 22, 8797–8807. 12388586 10.1523/JNEUROSCI.22-20-08797.2002 PMC6757677

[34] Taguchi, K., Watanabe, Y., Tsujimura, A. and Tanaka, M. (2019) Expression of α-synuclein is regulated in a neuronal cell type-dependent manner. Anat. Sci. Int., 94, 11–22. 30362073 10.1007/s12565-018-0464-8 PMC6315015

[35] Halliday, G.M., Holton, J.L., Revesz, T. and Dickson, D.W. (2011) Neuropathology underlying clinical variability in patients with synucleinopathies. Acta Neuropathol. (Berl.), 122, 187–204. 21720849 10.1007/s00401-011-0852-9

[36] Giguère, N., Burke Nanni, S. and Trudeau, L.-E. (2018) On cell loss and selective vulnerability of neuronal populations in Parkinson’s disease. Front. Neurol., 9, 455. 29971039 10.3389/fneur.2018.00455 PMC6018545

[37] Foffani, G. and Obeso, J.A. (2018) A cortical pathogenic theory of Parkinson’s disease. Neuron, 99, 1116–1128. 30236282 10.1016/j.neuron.2018.07.028

[38] Niccolini, F., Foltynie, T., Reis Marques, T., Muhlert, N., Tziortzi, A.C., Searle, G.E., Natesan, S., Kapur, S., Rabiner, E.A., Gunn, R.N. et al. (2015) Loss of phosphodiesterase 10A expression is associated with progression and severity in Parkinson’s disease. Brain J. Neurol., 138, 3003–3015. 10.1093/brain/awv219 26210536

[39] Halbgebauer, S., Nagl, M., Klafki, H., Haußmann, U., Steinacker, P., Oeckl, P., Kassubek, J., Pinkhardt, E., Ludolph, A.C., Soininen, H. et al. (2016) Modified serpinA1 as risk marker for Parkinson’s disease dementia: analysis of baseline data. Sci. Rep., 6, 26145. 27184740 10.1038/srep26145 PMC4868992

[40] Benkert, J., Hess, S., Roy, S., Beccano-Kelly, D., Wiederspohn, N., Duda, J., Simons, C., Patil, K., Gaifullina, A., Mannal, N. et al. (2019) Cav2.3 channels contribute to dopaminergic neuron loss in a model of Parkinson’s disease. Nat. Commun., 10, 5094. 31704946 10.1038/s41467-019-12834-x PMC6841684

[41] Date, I., Notter, M.F.D., Felten, S.Y. and Felten, D.L. (1990) MPTP-treated young mice but not aging mice show partial recovery of the nigrostriatal dopaminergic system by stereotaxic injection of acidic fibroblast growth factor (aFGF). Brain Res., 526, 156–160. 1706636 10.1016/0006-8993(90)90264-c

[42] Nishi, A. and Shuto, T. (2017) Potential for targeting dopamine/DARPP-32 signaling in neuropsychiatric and neurodegenerative disorders. Expert Opin. Ther. Targets, 21, 259–272. 28052701 10.1080/14728222.2017.1279149

[43] Schwarzschild, M.A., Agnati, L., Fuxe, K., Chen, J.-F. and Morelli, M. (2006) Targeting adenosine A2A receptors in Parkinson’s disease. Trends Neurosci., 29, 647–654. 17030429 10.1016/j.tins.2006.09.004

[44] Schwindinger, W.F., Mihalcik, L.J.M., Giger, K.E., Betz, K.S., Stauffer, A.M., Linden, J., Herve, D. and Robishaw, J.D. (2010) Adenosine A2A receptor Signaling and golf assembly show a specific requirement for the γ7 subtype in the striatum. J. Biol. Chem., 285, 29787–29796. 20639202 10.1074/jbc.M110.142620 PMC2943273

[45] Torres, E.R.S., Stanojlovic, M., Zelikowsky, M., Bonsberger, J., Hean, S., Mulligan, C., Baldauf, L., Fleming, S., Masliah, E., Chesselet, M.-F. et al. (2021) Alpha-synuclein pathology, microgliosis, and parvalbumin neuron loss in the amygdala associated with enhanced fear in the Thy1-aSyn model of Parkinson’s disease. Neurobiol. Dis., 158, 105478. 34390837 10.1016/j.nbd.2021.105478 PMC8447919

[46] Jiang, P., Gan, M., Yen, S.-H., McLean, P.J. and Dickson, D.W. (2017) Histones facilitate α-synuclein aggregation during neuronal apoptosis. Acta Neuropathol. (Berl.), 133, 547–558. 28004278 10.1007/s00401-016-1660-z PMC5350017

[47] Nishi, A., Kuroiwa, M., Miller, D.B., O’Callaghan, J.P., Bateup, H.S., Shuto, T., Sotogaku, N., Fukuda, T., Heintz, N., Greengard, P. et al. (2008) Distinct roles of PDE4 and PDE10A in the regulation of cAMP/PKA signaling in the striatum. J. Neurosci., 28, 10460–10471. 18923023 10.1523/JNEUROSCI.2518-08.2008 PMC2814340

[48] Fienberg, A.A., Hiroi, N., Mermelstein, P.G., Song, W.-J., Snyder, G.L., Nishi, A., Cheramy, A., O’Callaghan, J.P., Miller, D.B., Cole, D.G. et al. (1998) DARPP-32: regulator of the efficacy of dopaminergic neurotransmission. Science, 281, 838–842. 9694658 10.1126/science.281.5378.838

[49] Sasaki, K., Yamasaki, T., Omotuyi, I.O., Mishina, M. and Ueda, H. (2013) Age-dependent dystonia in striatal Gγ7 deficient mice is reversed by the dopamine D2 receptor agonist pramipexole. J. Neurochem., 124, 844–854. 23311775 10.1111/jnc.12149

[50] Ma, S.-X., Seo, B.A., Kim, D., Xiong, Y., Kwon, S.-H., Brahmachari, S., Kim, S., Kam, T.-I., Nirujogi, R.S., Kwon, S.H. et al. (2021) Complement and coagulation cascades are potentially involved in dopaminergic neurodegeneration in α-synuclein-based mouse models of Parkinson’s disease. J. Proteome Res., 20, 3428–3443. 34061533 10.1021/acs.jproteome.0c01002 PMC8628316

[51] Jung, S.Y., Choi, J.M., Rousseaux, M.W.C., Malovannaya, A., Kim, J.J., Kutzera, J., Wang, Y., Huang, Y., Zhu, W., Maity, S. et al. (2017) An anatomically resolved mouse brain proteome reveals Parkinson disease-relevant pathways^*^. Mol. Cell. Proteomics, 16, 581–593. 28153913 10.1074/mcp.M116.061440 PMC5383780

[52] Yger, M. and Girault, J.-A. (2011) DARPP-32, Jack of all trades… master of which? Front. Behav. Neurosci., 5, 56. 10.3389/fnbeh.2011.00056 PMC3168893 21927600

[53] Santini, E., Valjent, E., Usiello, A., Carta, M., Borgkvist, A., Girault, J.-A., Hervé, D., Greengard, P. and Fisone, G. (2007) Critical involvement of cAMP/DARPP-32 and extracellular signal-regulated protein kinase Signaling in l-DOPA-induced dyskinesia. J. Neurosci., 27, 6995–7005. 17596448 10.1523/JNEUROSCI.0852-07.2007 PMC6672217

[54] Dorsey, E.R., Sherer, T., Okun, M.S. and Bloem, B.R. The emerging evidence of the Parkinson pandemic. J. Parkinsons Dis., 8, S3–S8. 30584159 10.3233/JPD-181474 PMC6311367

[55] Bodén, R., Persson, J., Wall, A., Lubberink, M., Ekselius, L., Larsson, E.-M. and Antoni, G. (2017) Striatal phosphodiesterase 10A and medial prefrontal cortical thickness in patients with schizophrenia: a PET and MRI study. Transl. Psychiatry, 7, e1050–e1050. 28267149 10.1038/tp.2017.11 PMC5416662

[56] Abeliovich, A., Schmitz, Y., Fariñas, I., Choi-Lundberg, D., Ho, W.H., Castillo, P.E., Shinsky, N., Verdugo, J.M., Armanini, M., Ryan, A. et al. (2000) Mice lacking alpha-synuclein display functional deficits in the nigrostriatal dopamine system. Neuron, 25, 239–252. 10707987 10.1016/s0896-6273(00)80886-7

[57] Greten-Harrison, B., Polydoro, M., Morimoto-Tomita, M., Diao, L., Williams, A.M., Nie, E.H., Makani, S., Tian, N., Castillo, P.E., Buchman, V.L. et al. (2010) αβγ-Synuclein triple knockout mice reveal age-dependent neuronal dysfunction. Proc. Natl. Acad. Sci. U. S. A., 107, 19573–19578. 20974939 10.1073/pnas.1005005107 PMC2984188

[58] Burré, J., Sharma, M., Tsetsenis, T., Buchman, V., Etherton, M.R. and Südhof, T.C. (2010) α-Synuclein promotes SNARE-complex assembly in vivo and in vitro. Science, 329, 1663–1667. 20798282 10.1126/science.1195227 PMC3235365

[59] Anwar, S., Peters, O., Millership, S., Ninkina, N., Doig, N., Connor-Robson, N., Threlfell, S., Kooner, G., Deacon, R.M., Bannerman, D.M. et al. (2011) Functional alterations to the nigrostriatal system in mice lacking all three members of the synuclein family. J. Neurosci., 31, 7264–7274. 21593311 10.1523/JNEUROSCI.6194-10.2011 PMC3154647

[60] Timney, B.L., Raveh, B., Mironska, R., Trivedi, J.M., Kim, S.J., Russel, D., Wente, S.R., Sali, A. and Rout, M.P. (2016) Simple rules for passive diffusion through the nuclear pore complex. J. Cell Biol., 215, 57–76. 27697925 10.1083/jcb.201601004 PMC5057280

[61] Siddiqui, A., Chinta, S.J., Mallajosyula, J.K., Rajagopolan, S., Hanson, I., Rane, A. and Andersen, J.K. (2012) Selective binding of nuclear alpha-synuclein to the PGC1alpha promoter under conditions of oxidative stress may contribute to losses in mitochondrial function: implications for Parkinson’s disease. Free Radic. Biol. Med., 53, 993–1003. 22705949 10.1016/j.freeradbiomed.2012.05.024 PMC3418424

[62] Jiang, K., Rocha, S., Westling, A., Kesarimangalam, S., Dorfman, K.D., Wittung-Stafshede, P. and Westerlund, F. (2018) Alpha-synuclein modulates the physical properties of DNA. Chem. Weinh. Bergstr. Ger., 24, 15685–15690. 10.1002/chem.201803933 PMC6217799 30102440

[63] Vasudevaraju, P., Guerrero, E., Hegde, M.L., Collen, T.B., Britton, G.B. and Rao, K.S. (2012) New evidence on α-synuclein and tau binding to conformation and sequence specific GC^*^ rich DNA: relevance to neurological disorders. J. Pharm. Bioallied Sci., 4, 112–117. 22557921 10.4103/0975-7406.94811 PMC3341714

[64] Ma, K.-L., Song, L.-K., Yuan, Y.-H., Zhang, Y., Yang, J.-L., Zhu, P. and Chen, N.-H. (2014) α-Synuclein is prone to interaction with the GC-box-like sequence in vitro. Cell. Mol. Neurobiol., 34, 603–609. 24659023 10.1007/s10571-014-0046-9 PMC11488944

[65] Rousseaux, M.W., Revelli, J.-P., Vázquez-Vélez, G.E., Kim, J.-Y., Craigen, E., Gonzales, K., Beckinghausen, J. and Zoghbi, H.Y. (2018) Depleting Trim28 in adult mice is well tolerated and reduces levels of α-synuclein and tau. eLife, 7, e36768. 29863470 10.7554/eLife.36768 PMC5993537

[66] Deacon, R.M.J. (2006) Assessing nest building in mice. Nat. Protoc., 1, 1117–1119. 17406392 10.1038/nprot.2006.170

[67] Tokarew, J.M., El-Kodsi, D.N., Lengacher, N.A., Fehr, T.K., Nguyen, A.P., Shutinoski, B., O’Nuallain, B., Jin, M., Khan, J.M., Ng, A.C.H. et al. (2021) Age-associated insolubility of parkin in human midbrain is linked to redox balance and sequestration of reactive dopamine metabolites. Acta Neuropathol. (Berl.), 141, 725–754. 33694021 10.1007/s00401-021-02285-4 PMC8043881

[68] Perez-Riverol Y, Bai J, Bandla C, Hewapathirana S, García-Seisdedos D, Kamatchinathan S, Kundu D, Prakash A, Frericks-Zipper A et al. (2022) The PRIDE database resources in 2022: A Hub for mass spectrometry-based proteomics evidences. Nucleic Acids Res., 50, D543–D552. 34723319 10.1093/nar/gkab1038 PMC8728295

