# Supplementary figures and images for: Constitutive nuclear accumulation of endogenous alpha-synuclein in mice causes motor impairment and cortical dysfunction, independent of protein aggregation
Source: Hum Mol Genet. 2022 Feb 18;31(21):3613–28. doi: 10.1093/hmg/ddac035 (PMC9616578; doi:10.1093/hmg/ddac035)

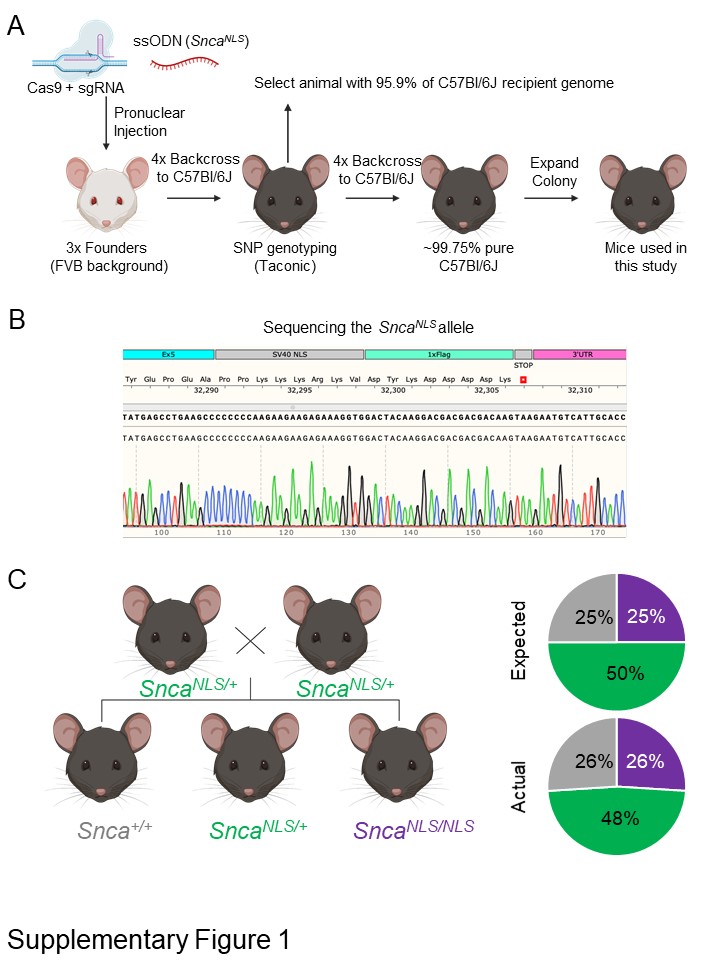

Supplement: Supplementary_Figure_1_ddac035 [file supplementary_figure_1_ddac035.jpeg]

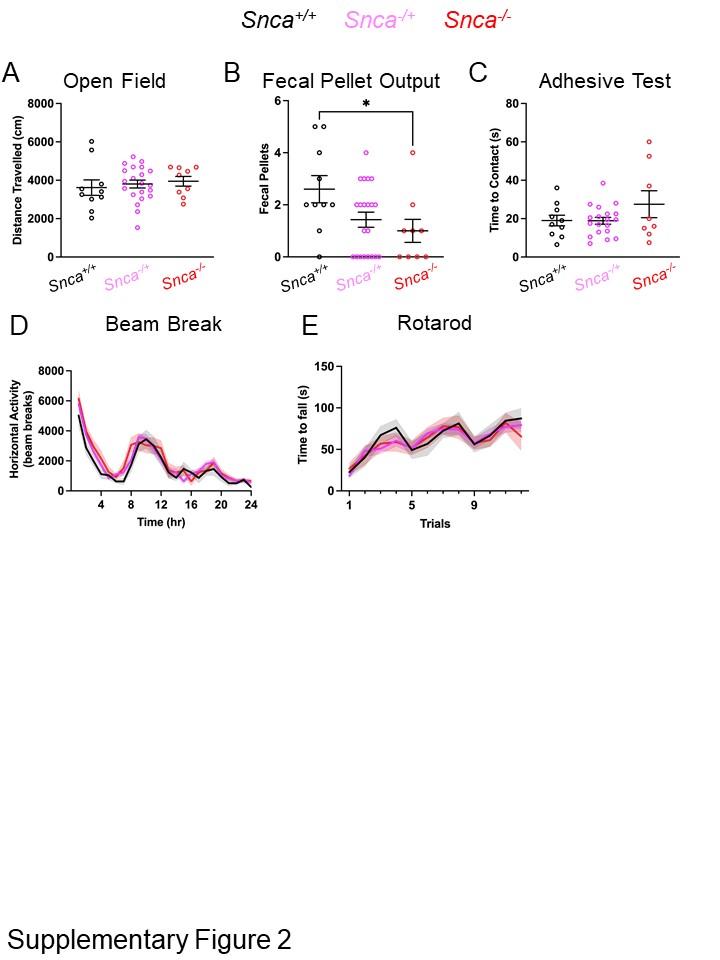

Supplement: Supplementary_Figure_2_ddac035 [file supplementary_figure_2_ddac035.jpeg]

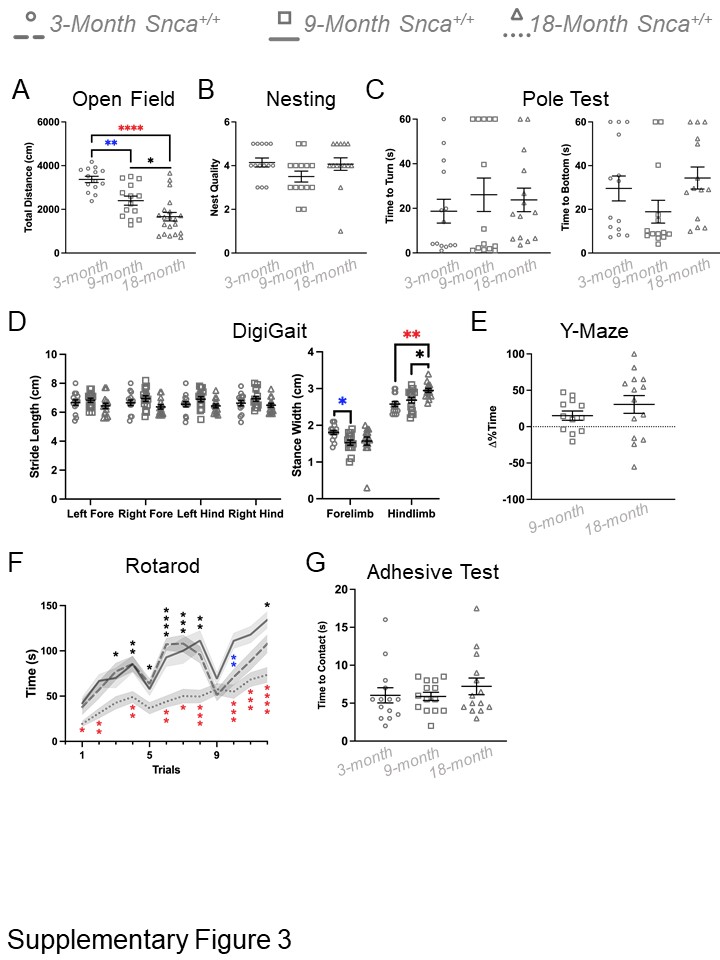

Supplement: Supplementary_Figure_3_ddac035 [file supplementary_figure_3_ddac035.jpeg]

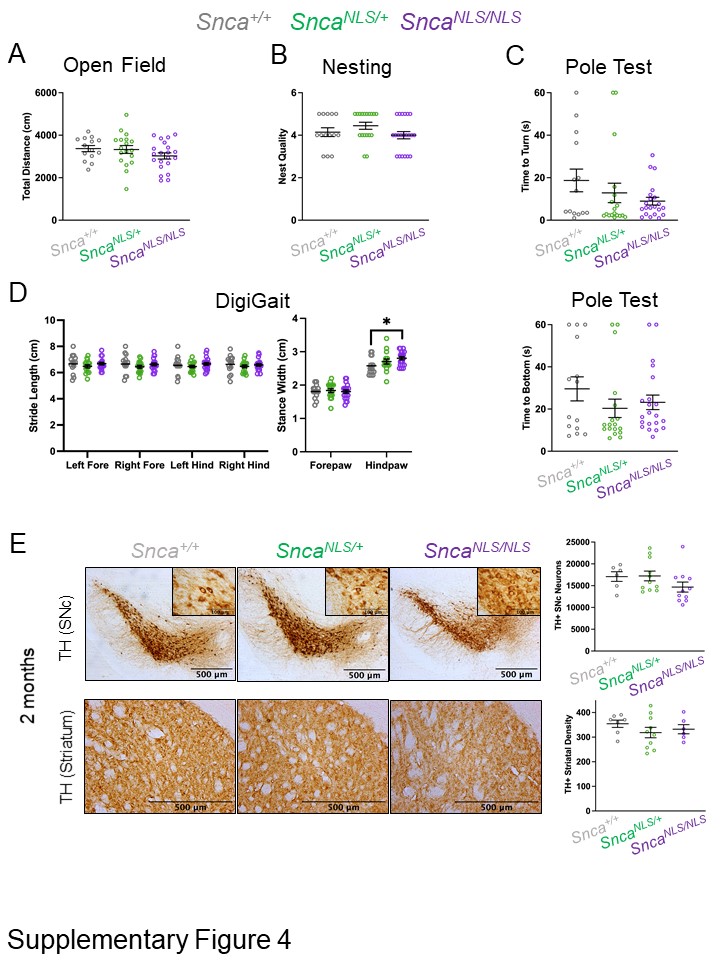

Supplement: Supplementary_Figure_4_ddac035 [file supplementary_figure_4_ddac035.jpeg]

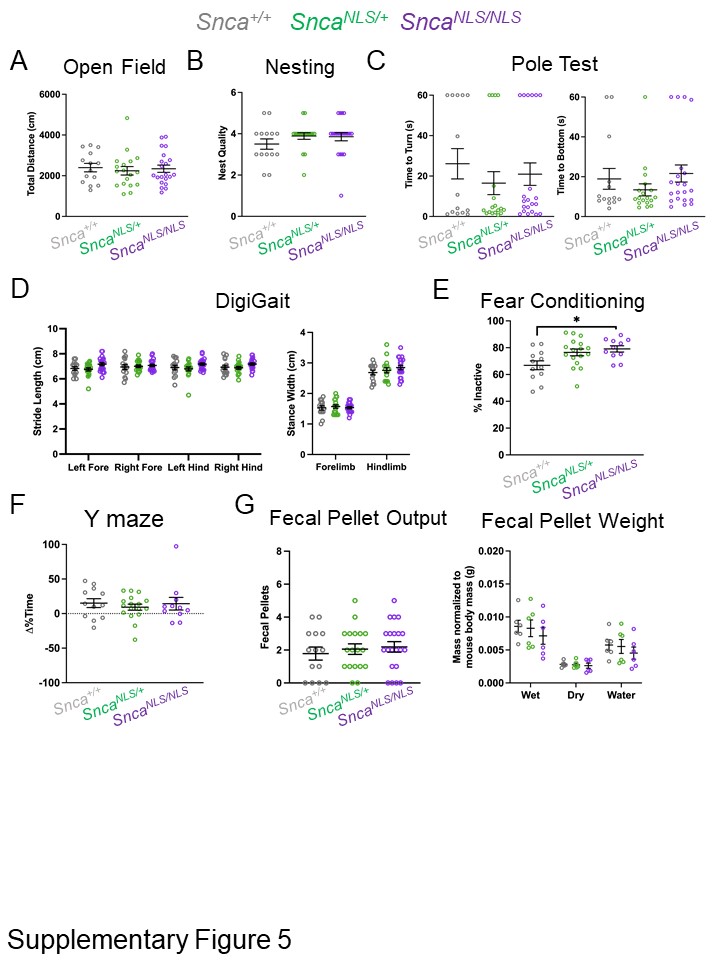

Supplement: Supplementary_Figure_5_ddac035 [file supplementary_figure_5_ddac035.jpeg]

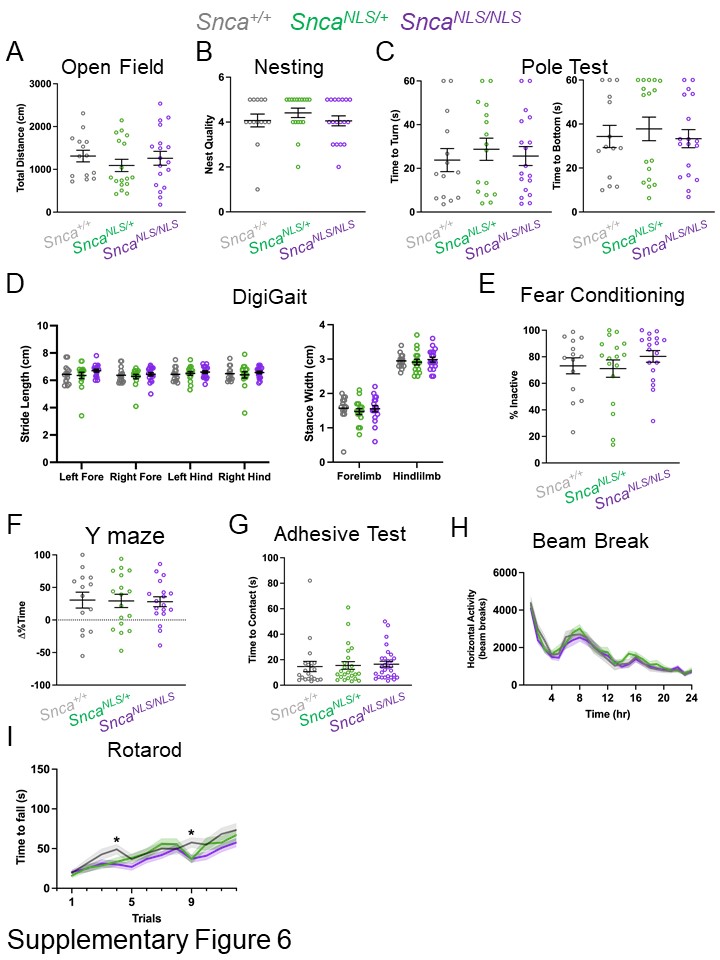

Supplement: Supplementary_Figure_6_ddac035 [file supplementary_figure_6_ddac035.jpeg]

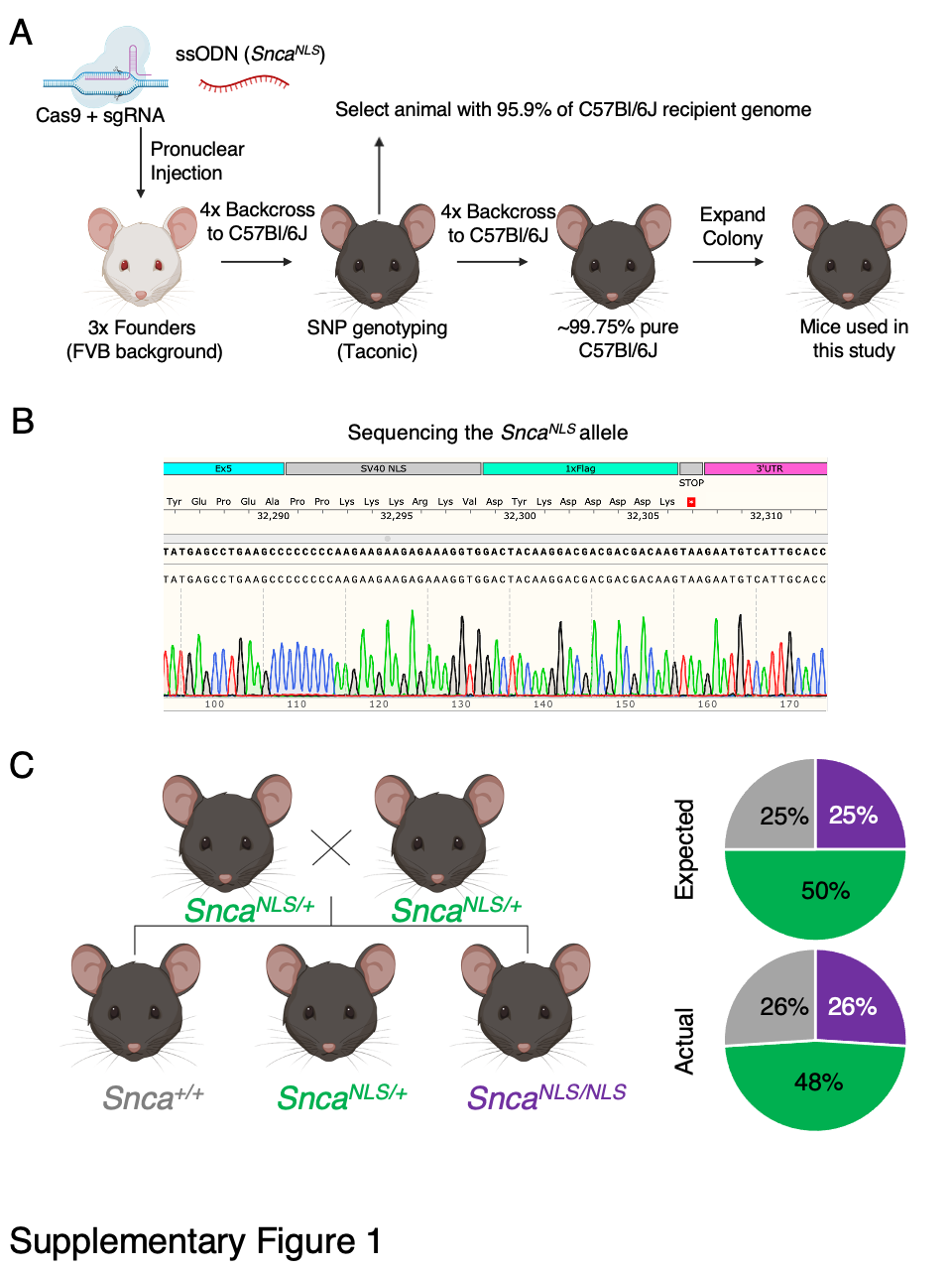

Supplement: Slide8_ddac035 [file slide8_ddac035.zip › Slide8_ddac035.tiff]

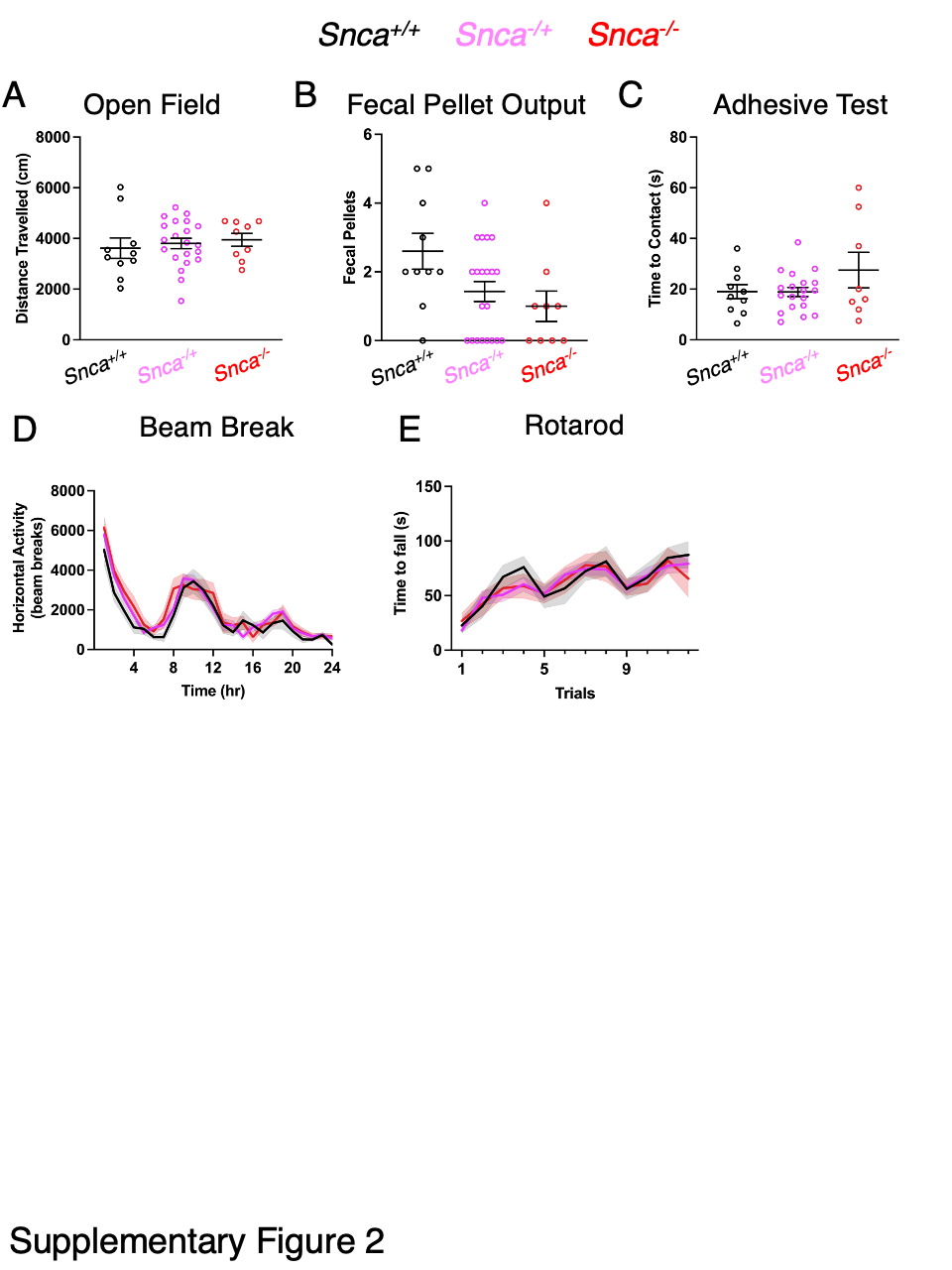

Supplement: Slide9_ddac035 [file slide9_ddac035.zip › Slide9_ddac035.tiff]

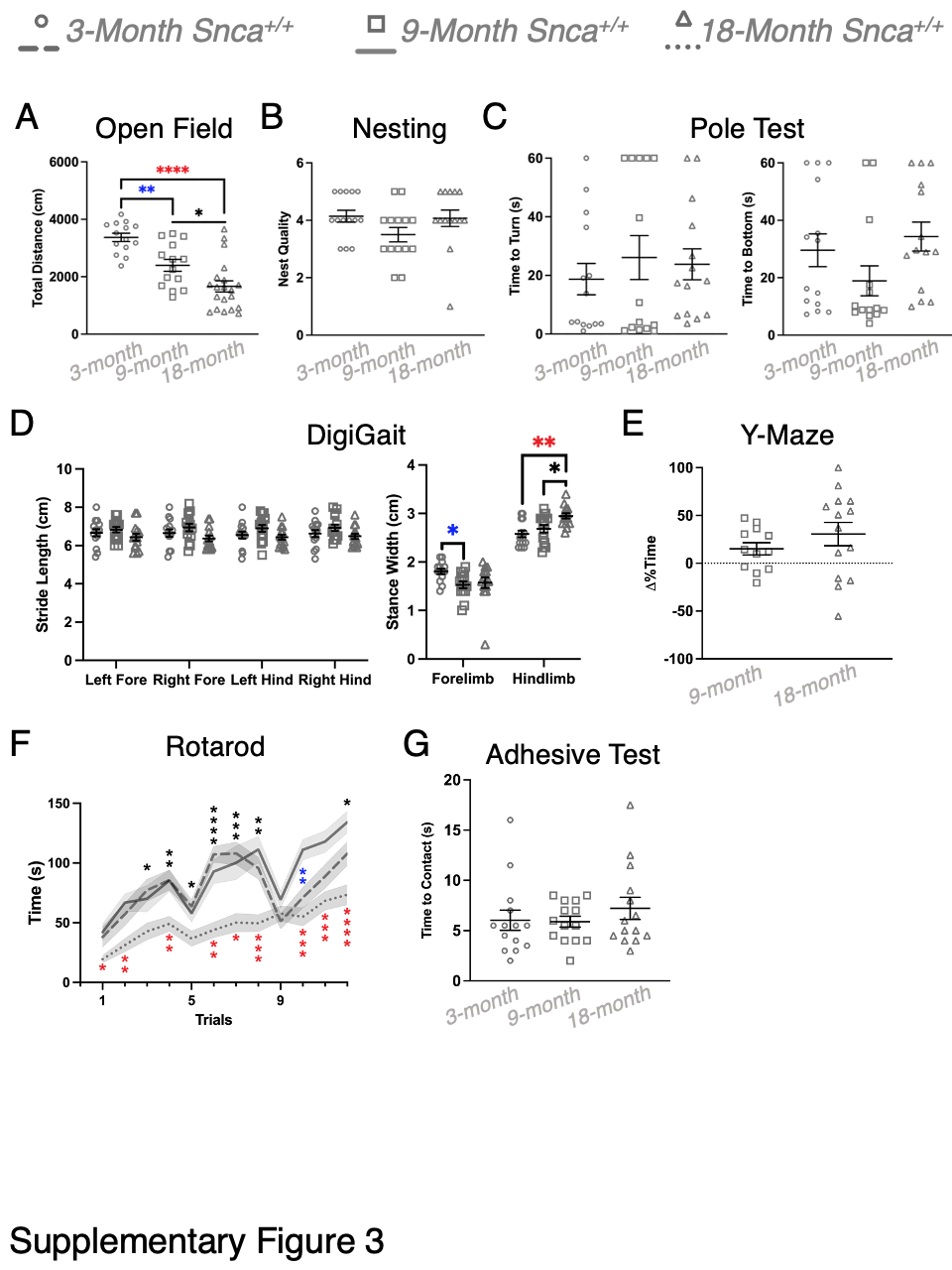

Supplement: Slide10_ddac035 [file slide10_ddac035.zip › Slide10_ddac035.tiff]

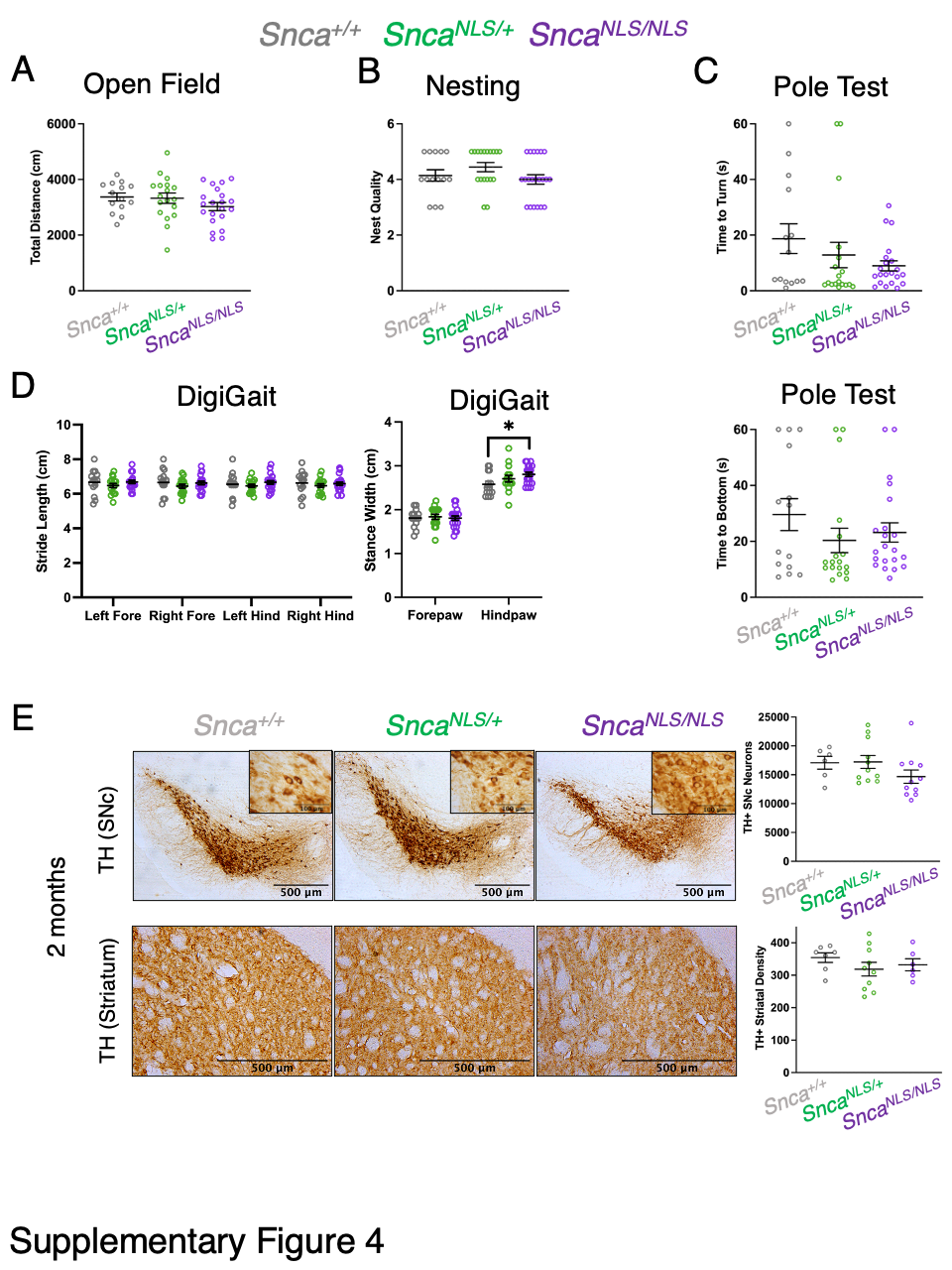

Supplement: Slide11_ddac035 [file slide11_ddac035.zip › Slide11_ddac035.tiff]

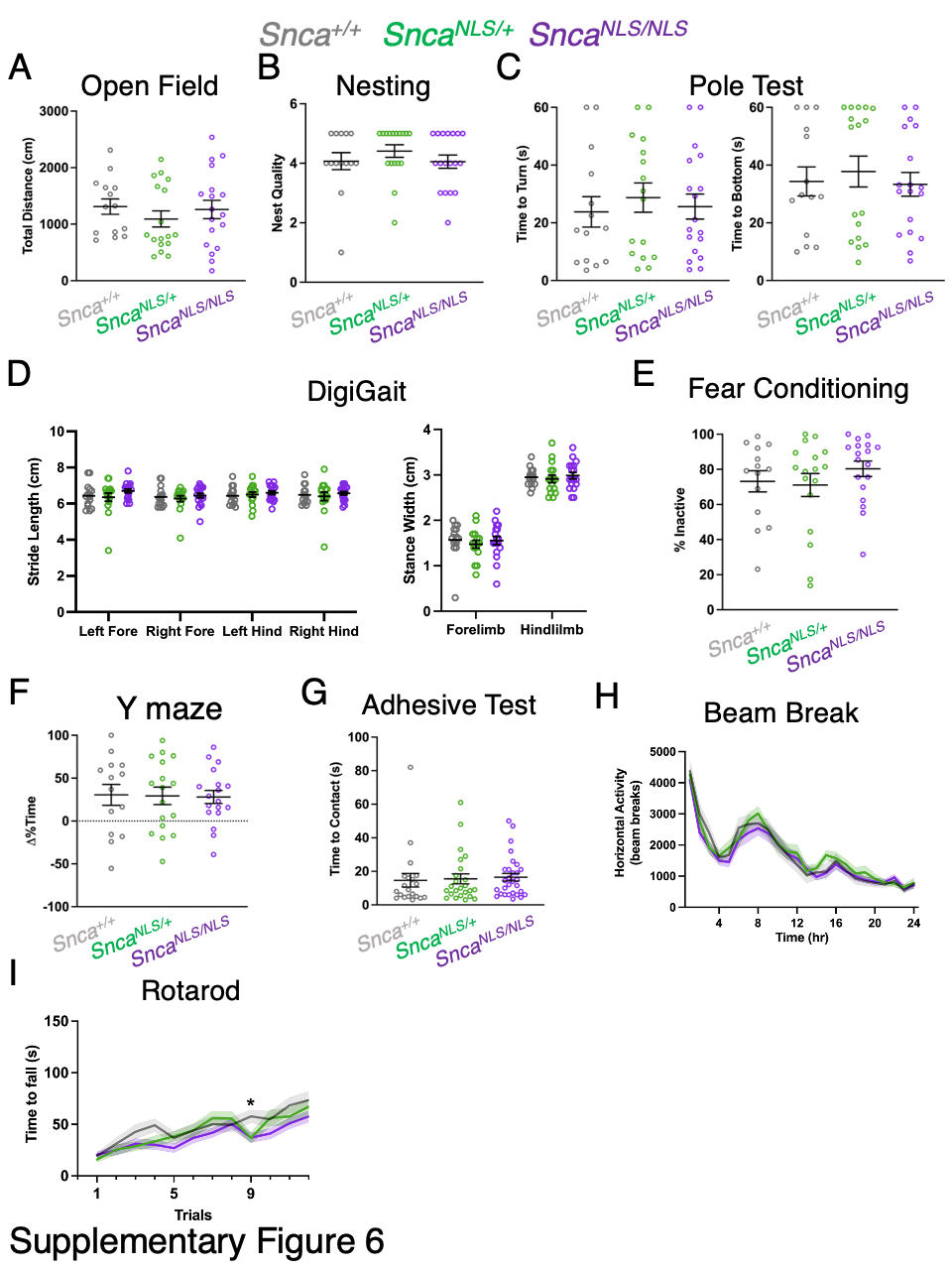

Supplement: Slide13_ddac035 [file slide13_ddac035.zip › Slide13_ddac035.tiff]
