# Supplementary material for: Constitutive nuclear accumulation of endogenous alpha-synuclein in mice causes motor impairment and cortical dysfunction, independent of protein aggregation
Source: Hum Mol Genet. 2022 Feb 18;31(21):3613–28. doi: 10.1093/hmg/ddac035 (PMC9616578; doi:10.1093/hmg/ddac035)
Supplement: supplementary_legends_ddac035 [file supplementary_legends_ddac035.docx]

**Constitutive nuclear accumulation of endogenous alpha-synuclein in mice causes motor impairment and cortical dysfunction, independent of protein aggregation.**

Haley M. Geertsma^1,2^, Terry R. Suk^1,2^, Konrad M. Ricke^1,2,3^, Kyra Horsthuis^1,2^, Jean-Louis A. Parmasad^1,2^, Zoe A. Fisk^1,2,3^, Steve M. Callaghan^1,2^, Maxime W.C. Rousseaux^1,2,3,4,5*^

Supplementary legends:

**Supplementary Figure 1: Generation of the *Snca^NLS^* mice.** A) *Snca^NLS^* mouse generation. B) Sequencing confirms the presence of the knock-in. C) Breeding scheme with expected and actual Mendelian ratios among offspring. n = 148 *Snca^+/+^*, n=272 *Snca^NLS/+^*, and n=147 *Snca^NLS/NLS^*. Chi squared test: *P* = 0.7028.

**Supplementary Figure 2: Comprehensive behavior profiling of 9-month-old *Snca^-/-^* mice compared to littermates.** Behavior analysis of 9-month *Snca^-/-^* mice included (A) Open Field, (B) Fecal Pellet Output Test, (C) Adhesive Test, (D) Beam Break measuring horizontal activity, and (E) Rotarod (n=9-21). One‑ (A,B,C) or Two-Way ANOVA (D,E) with Bonferroni multiple comparison: ns, * denotes *P* > 0.05 and < 0.05, respectively.

**Supplementary Figure 3:** **Wild-type mice exhibit age-dependent decline in motor ability.** Behavior comparison of wild-type mice at 3-, 9-, and 18-months in (A) Open Field, (B) Nesting, (C) Pole test measuring time to turn over (left) and time to descend to the bottom (right), (D) Digigait measuring stride length (left) and stance width (right), and (E) Y maze (n = 14-20). Motor assays with additional cohorts of mice include (F) Rotarod and (G) Adhesive Test measuring time to contact their forepaws (n = 9-20). One- (A,B,C,E,G) or Two-Way ANOVA (D,F) with Bonferroni multiple comparison: ns, *, **, ***, and **** denote *P* > 0.05, < 0.05, < 0.01, < 0.001, and < 0.0001, respectively. Blue asterisk denotes significance between 3- and 9-month mice, black asterisk denotes significance between 9- and 18-month mice, and red asterisk denotes significance between 3- and 18-month mice.

**Supplementary Figure 4: Comprehensive behavior and dopaminergic profiling of young *Snca^NLS/NLS^* mice.** Behavior analysis of 3-month mice in (A) Open Field, (B) Nesting, (C) Pole test measuring time to turn over (upper) and time to descend to the bottom (lower), and (D) DigiGait measuring stride length (left) and stance width (right) (n = 14-21). E) Tyrosine hydroxylase staining of the Substantia nigra *pars compacta* (upper) and striatum (lower) of 2-month mice with their respective quantifications (right) (n = 6-11). One- (A,B,C,E) or Two- Way ANOVA (D) with Bonferroni multiple comparison: ns, * denote *P* > 0.05 and < 0.05, respectively.

**Supplementary Figure 5: Comprehensive behavior profiling of 9-month-old *Snca^NLS/NLS^* mice compared to littermates.** Behavior analysis of 9-month mice in motor assays including (A) Open Field, (B) Nesting, (C) Pole test measuring time to turn over (left) and time to descend to the bottom (right), and (D) DigiGait measuring stride length (left) and stance width (right). Non-motor assays include (E) Fear conditioning, (F) Y maze forced alteration, and (G) Fecal pellet production measuring fecal output in 10 minutes (left) and the weight and water content of fecal pellets produced over 1 hour (right) (n = 14-21). One- (A,B,C,E,F,G left) or Two-Way ANOVA (D,G right) with Bonferroni multiple comparison: ns, * denote *P* > 0.05 and < 0.05, respectively.

**Supplementary Figure 6: Comprehensive behavior profiling of 18-month-old *Snca^NLS/NLS^* mice compared to littermates.** Behavior analysis of 18-month mice in motor assays including (A) Open Field, (B) Nesting, (C) Pole test measuring time to turn over (left) and time to descend to the bottom (right), and (D) Digigait measuring stride length (left) and stance width (right). Non-motor assays include (E) Fear conditioning and (F) Y maze (n = 14-18). Motor assays with additional cohorts of mice include (G) Adhesive Test measuring time to contact their forepaws, (H) Beam Break measuring horizontal activity, and (I) Rotarod (n = 20-31). One- (A,B,C,E,F,G) or Two-Way ANOVA (D,H,I) with Bonferroni multiple comparison: ns, * denote *P* > 0.05 and < 0.05, respectively.

**Tables:**

**Supplementary Table 1: List of antibodies used throughout the study**

**Supplementary Table 2: List of hits from proteomic profiling of cortices from 9-month-old *Snca^NLS/NLS^* mice compared to wild-type littermates**
